# Changes in Biomechanical Properties of A375 Cells Due to the Silencing of *TMSB4X* Expression Are Not Directly Correlated with Alterations in Their Stemness Features

**DOI:** 10.3390/cells10040769

**Published:** 2021-03-31

**Authors:** Aleksandra Makowiecka, Ewa Mazurkiewicz, Ewa Mrówczyńska, Natalia Malek, Alice Battistella, Marco Lazzarino, Dorota Nowak, Antonina Joanna Mazur

**Affiliations:** 1 Department of Cell Pathology, Faculty of Biotechnology, University of Wroclaw, 50-383 Wrocław, Poland; ewa.mazurkiewicz@uwr.edu.pl (E.M.); ewa.mrowczynska@uwr.edu.pl (E.M.); en.malek@gmail.com (N.M.); dorota.nowak@uwr.edu.pl (D.N.); 2 Istituto Officina dei Materiali-National Research Council, I-34149 Trieste, Italy; battistella@iom.cnr.it (A.B.); lazzarino@iom.cnr.it (M.L.)

**Keywords:** Thymosin β4 (Tβ4), F-actin, Vimentin, Nestin, cytoskeleton, stemness, tumorigenicity, clonogenicity, melanoma, melanoma heterogeneity, phenotypic heterogeneity, stiffness, atomic force microscopy (AFM), single-cell force spectroscopy (SFCS)

## Abstract

Thymosin β4 (Tβ4) is a small, 44-amino acid polypeptide. It has been implicated in multiple processes, including cell movement, angiogenesis, and stemness. Previously, we reported that melanoma cell lines differ in Tβ4 levels. Studies on stable clones with silenced *TMSB4X* expression showed that Tβ4 impacted adhesion and epithelial-mesenchymal transition progression. Here, we show that the cells with silenced *TMSB4X* expression exhibited altered actin cytoskeleton’s organization and subcellular relocalization of two intermediate filament proteins: Nestin and Vimentin. The rearrangement of the cell cytoskeleton resulted in changes in the cells’ topology, height, and stiffness defined by Young’s modulus. Simultaneously, only for some A375 clones with a lowered Tβ4 level, we observed a decreased ability to initiate colony formation in soft agar, tumor formation in vivo, and alterations in Nanog’s expression level transcription factor regulating stemness. Thus, we show for the first time that in A375 cells, biomechanical properties are not directly coupled to stemness features, and this cell line is phenotypically heterogeneous.

## 1. Introduction

Thymosin β4 (Tβ4) belongs to the β thymosin family of structurally related, hormone-like, highly conserved polypeptides [1]. Tβ4 was identified as the significant sequestering agent of monomeric actin (G-actin) in mammalian cells, directly involved in the actin cytoskeleton’s reorganization during cell movement [2]. It plays an essential and complex role in wound healing: migration of keratinocytes and endothelial cells [3], stimulation of angiogenesis [4], recruiting and supporting the differentiation of progenitor cells at the injury site [5], reducing inflammation [6], and immunomodulatory activity [7]. These properties of Tβ4 raised questions about its role in cancer development and progression. Despite research conducted on various types of cancer cells, the role of this polypeptide in tumorigenesis remains unclear. Our previous study showed that Tβ4 in human melanoma cells is a component of focal adhesion (FA) and plays a role in forming this structure [8]. The cells with low *TMSB4X* (gene encoding Tβ4) expression levels formed a higher number of FAs than the cells with high *TMSB4X* expression levels. However, these FAs were of a smaller size than those observed in the cells with a high Tβ4 synthesis level. Additionally, we observed that the silencing of *TMSB4X* expression in melanoma cells affected their adhesion, migration, and invasion abilities.

Wirsching with colleagues observed that lowering Tβ4′s level in glioblastoma cells decreased those cells’ invasive potential [9]. Moreover, the same study showed that *TMSB4X* expression silencing led to decreased stemness of glioblastoma cells. In tumors and established cancer cell lines, there is a subpopulation of cells manifesting stem cell features—cancer stem cells (CSCs), also called tumor-initiating cells [10]. CSCs are characterized by some standard features, i.e., self-renewal, differentiation to other types of tumor cells, resistance to chemotherapy, and radiotherapy [11]. In 2005, the presence of CSC cell populations was reported in melanoma cell lines [12] and, in 2008, in tumor samples collected from patients [13]. In recent years, an increasing number of data has been published presenting the involvement of Thymosin β4 in cell stemness and differentiation. In neural stem cells, downregulation of Tβ4 expression promoted their differentiation [14]. Colon cancer stem cells obtained from different patients exhibited higher Tβ4 levels than normal epithelial cells [15]. Moreover, the downregulation of Tβ4 in these cells impaired their tumorigenic activity. Also in ovarian cancers, a high Tβ4 expression level was detected for CSCs [16]. Increased Tβ4 expression in adipose-derived mesenchymal stem cells was positively correlated with ovarian cancer’s metastasis [17].

A growing series of data implicates the actin cytoskeleton and mechanical properties in stem cell differentiation [18]. It has been shown that induced pluripotent stem cells (iPSCs) have a less developed cytoskeleton than fibroblasts [19]. A colony of human iPSCs cells was characterized by thick ventral stress fibers forming “an actin fence.” In contrast, paternal fibroblasts were characterized by multiple thin actin stress fibers aligned across the cell body. Mechanical phenotyping of mouse embryonic stem cells (mESC) using atomic force microscopy (AFM) revealed significant differences in their stiffness defined by Young’s modulus [20]. Early differentiated (6 days) mESCs cells were 3 times stiffer than those undifferentiated due to the increasing filamentous actin density and alterations in nuclear cytoskeleton composition. It has been demonstrated for various tumor types that the cells with higher metastatic potential exhibited decreased stiffness [21,22,23].

In our research, we evaluated the contribution of Tβ4 in melanoma cell cytoskeleton organization and mechanical properties in correlation to their stemness features in the meaning of tumorigenic and clonogenic potentials [24]. For this purpose, we conducted experiments on A375 cells with a lowered level of Tβ4 (sh-Tβ4 clones) obtained using shRNAs. These stable clones were characterized in our previous paper [8]. Additionally, we examined two melanoma cell lines: A375 and WM1341D, in which endogenous levels of Tβ4 are, respectively, high and low. According to our knowledge, no studies were published so far trying to uncover the interplay between mechanics and stemness in melanoma cells regarding the Tβ4 level. We show that Tβ4 in melanoma cells regulates the cells’ stiffness, most probably due to affecting actin cytoskeleton and intermediate filaments (IFs) organization. Additionally, we observed diminished clonality and tumorigenicity for the cells with less Tβ4; however for only a subset of clones. This, together with varying expression levels of transcription factors, implicated in stemness depending on analyzed clone imply that we observed phenotypic heterogeneity of A375 cells. We propose that Tβ4 by acting on the organization of actin cytoskeleton and intermediate filaments influences biomechanical properties and only partly stemness of A375 cells. Altogether we show for the first time that these parameters are not directly interconnected.

## 2. Materials and Methods

### 2.1. Cell Lines and Culture Conditions

The A375 cell line was obtained from the ATCC (ATCC^®^ CRL-1619™). The WM1341D cell line was from the Rockland Immunochemicals and is described elsewhere [25]. The procedures for obtaining and analyzing stable clones with decreased *TMSB4X* expression (sh-Tβ4 clones) and control cells (scr) were described elsewhere [8]. In brief, plasmids for *TMSB4X* gene silencing were obtained from Sigma-Aldrich (Poznań, Poland)—five MISSION pLKO.1-puro *TMSB4X* shRNA vectors and a control MISSION pLKO.1 puro Non-Target shRNA. The sequences are listed in the afore-cited publication in the Supplementary Data [8]. The cells were electroporated to introduce plasmids to the cells. All five sh-Tβ4 clones were obtained by electroporation with a mixture of all five *TMSB4X* shRNA sequences. All clones were used in all experiments and then merged into the “sh-Tβ4” group. The control “scr” cells were produced similarly, but a control plasmid was used. The results shown separately for every clone are presented in the Supplementary Material. Dulbecco’s modified Eagle’s medium with reduced concentration (1.5 g/L) of NaHCO_3_, Supplemented with 10% FBS, 1% L-Glutamine, and 1% Antibiotic-Antimycotic from Thermo Fisher Scientific (Warsaw, Poland) was used. The stable A375 cell clones were cultured in a medium with the addition of 1 µg/mL puromycin from Santa Cruz Biotechnology Inc. (Heidelberg, Germany). Cells were cultivated at 37 °C under a humidified atmosphere of 5% CO_2_ and subcultured twice a week.

### 2.2. The Soft Agar Colony Formation Assay

The assay was performed as described elsewhere [26]. Serum-free stem cell medium (SCM) was used: 1:1 Dulbecco’s Modified Eagle Medium/Nutrient Mixture F-12 (Thermo Fisher Scientific, Warsaw, Poland), 1x B-27 Supplement (Thermo Fisher Scientific, Warsaw, Poland), 10 ng/mL bFGF (Peprotech, London, UK), 20 ng/mL EGF (Merck, Darmstadt, Germany), 10 μg/mL insulin (Merck, Darmstadt, Germany), 1 ng/mL heparin (Thermo Fisher Scientific, Warsaw, Poland), and 1% Antibiotic-Antimycotic (Thermo Fisher Scientific, Warsaw, Poland). The cells were cultivated for 21 days in an atmosphere of 5% CO_2_ at 37 °C with SCM medium Supplementation onto the agar surface every 2–3 days. Colonies were stained with 0.005% crystal violet (Merck, Darmstadt, Germany). Photos were taken using an Olympus FluoView 500 confocal microscope. The number of colonies and their size was estimated using ImageJ (ImageJ, version 1.52p, F. Cordelieres, Institute Curie, Paris, France). The number of colonies and their area was calculated based on 21 microscopic pictures for every condition obtained from three separate experiments.

### 2.3. Chorioallantoic Membrane (CAM) Assay

The CAM metastasis assay was performed based on the procedure described elsewhere [27]. Briefly, fertilized chicken eggs were incubated in an incubator at 37 °C and with 80% humidity for 7 days. On day 7, eggs were windowed to detach the CAM membrane from an egg’s shell and expose the allantoic vein under aseptic conditions. Meanwhile, the cells’ solutions were prepared in a 40 × 10^6^ cells/mL concentration in a serum-free medium. Then 1 × 10^6^ cells were grafted on the CAM of each egg. After sealing the window in the egg’s shell with tape, all eggs were incubated for 7 days to allow the cells to form primary tumors. On day 14, both the percent and the area of developed tumors were determined. The cells were engrafted on 33 and 38 embryos in scr and sh-Tβ4 cells, respectively. The size of tumors was measured for all formed tumors (*n* = 17) for both types of clones. Photos of tumors were taken using a fluorescence stereomicroscope Leica M205 and Leica Application Suite (4.12.0) software. Analysis of the tumors’ area was performed manually using the “freehand selection” tool of the Fiji application [28].

### 2.4. Ethics Statement

Experiments with the use of chicken embryos were performed following Polish and European acts on the Protection of Animals Used for Scientific or Educational Purposes. No formal permits were required to carry out these experiments. All procedures were performed using the minimum number of embryos and minimizing any possible embryos’ suffering.

### 2.5. Quantitative Polymerase Chain Reaction (qPCR) and RT-PCR

RNA was isolated from the cells growing in 6-wells plates using GenElute™ Mammalian Total RNA Miniprep Kit (Merck, Darmstadt, Germany). Subsequently, the DNA was digested with DNase I (Merck, Darmstadt, Germany) according to the manufacturer’s protocol. Total RNA (0.5 μg) was reversed transcribed to cDNA using a High Capacity cDNA Reverse Transcription Kit (Thermo Fisher Scientific, Warsaw, Poland). PowerUp™ SYBR™ Green Master Mix (Thermo Fisher Scientific, Warsaw, Poland) and Applied Biosystems StepOne™ were used to perform qPCR reactions according to the manufacturer’s protocol. *HPRT1* gene was used for normalization. Following primers were used *HPRT1*; fwd: 5′GACCAGTCAACAGGGGACAT3′, rev: 5′GCTTGCGACCTTGACCATCT3′, *TMSB4X*; fwd: 5′GACAGAGACGCAAGAGAAAAATC3′, rev: 5′CGCCAATATGCACTGTACATTCC3′, *MYC*: fwd: 5′CTCGGATTCTCTGCTCTCCTC3′, rev: 5′GAGGTTTGCTGTGGCCTCCA3′, *NANOG*: fwd: 5′GATAGATTTCAGAGACAGAAATACC3′, rev: 5′GATTTCATTCTCTGGTTCTGGAAC3′; *NES*: fwd: 5′GTAGCTCCCAGAGAGGGGAA, rev: 5′CTCTAGAGGGCCAGGGACTT3′; *SOX2*: fwd: 5′AACCAGCGCATGGACAGTTA3′, rev: 5′GACTTGACCACCGAACCCAT3′.

For RT-PCR analysis 1 μL of cDNA was taken as a template, starters for *HPRT1* and *TMSB4X* at a final concentration of 500 nM, and Color Taq PCR Master Mix (2x) (EurX, Gdańsk, Polska). Polymerase chain reaction (25 cycles) was performed accordingly to the manufacturer’s protocol. The PCR products were following run on 2% agarose gels in Tris-acetate-EDTA (TAE) buffer and visualized with ChemiDocTM MP System and ImageLab 4.0 software, both from Bio-Rad (Hercules, CA, USA). GeneRuler 100 bp Plus DNA Ladder from Thermo Fisher Scientific (Warsaw, Poland) was used as a marker.

### 2.6. Immunocytochemistry and Confocal Microscopy

Immunocytochemical analyses were performed as described elsewhere [29]. Cells cultured in complete medium for 24 h on coverslips were fixed with 4% formaldehyde in phosphate-buffered saline (PBS), permeabilized with 0.1% Triton X-100 in PBS, and blocked with 1% bovine serum albumin (BSA) in PBS solution. The coverslips were incubated overnight at 4 °C with primary antibodies diluted in blocking solution. We used as primary antibodies mouse anti-Nestin (10c2) and rabbit anti-Emerin (FL-254) from Santa Cruz Biotechnology Inc. (Heidelberg, Germany) in 1:50 dilution, rabbit anti-Vimentin (D21H3) from Cell Signaling Technology (Beverly, MA, USA) in 1:100 dilution, and mouse anti-Vimentin (V9) from Sigma-Aldrich (Poznań, Poland) in 1:1000 dilution. In the next step, the following secondary antibodies were used: donkey anti-mouse-Alexa Fluor 488, donkey anti-rabbit-Alexa Fluor 488, or 647 from Thermo Fisher Scientific (Warsaw, Poland). The dilution of secondary antibodies was 1:200. We detected monomeric and filamentous actin by using, respectively, deoxyribonuclease I-Alexa Fluor 594, phalloidin-Alexa Fluor 568, or phalloidin-Alexa Fluor 488 in 1:100 dilution. For detection of the cell nucleus, Hoechst 33342 in 1:1000 dilution was used. Secondary antibodies and dyes were from Thermo Fisher Scientific (Warsaw, Poland). The coverslips were then mounted on microscopic glass with Dako Mounting Medium from Agilent Technologies Inc. (Santa Clara, CA, USA). Photos were taken using the Leica TCS SP8 Confocal Laser Scanning Microscope and followingly analyzed with the Leica Application Suite X (LAS X) software.

### 2.7. Western Blot Analysis (WB)

Cell lysates were prepared on ice using cytoskeletal-bound protein extraction buffer (CB): 10 mM Tris-HCl pH 7.4, 100 mM NaCl, 1 mM EDTA, 1 mM EGTA, 1 mM NaF, 20 mM Na_4_P_2_O_7_, 2 mM Na_3_VO_4_, 1% Triton X-100, 10% glycerol, 0.1% SDS, 0.5% sodium deoxycholate with addition of 1:100 protease inhibitors cocktail (Merck, Darmstadt, Germany). Protein concentration was determined using the Bradford protein assay (Merck, Darmstadt, Germany). Samples containing 30 μg of protein were separated in 12.5% polyacrylamide gel by SDS-PAGE and transferred to nitrocellulose using a wet transfer method. Transfer efficiency and control analysis of protein loading utilized Ponceau S membrane staining. Depending on the antibody manufacturer’s recommendations, membranes were blocked in either 5% skimmed milk or 5% BSA solution in TBS-T (50 mM Tris-HCl pH 7.6, 150 mM NaCl, and 0.1% Tween 20). The following primary antibodies were used: mouse anti-Nestin (10c2), mouse anti-c-Myc (9e10) in 1:200 dilution were from Santa Cruz Biotechnology Inc. (Heidelberg, Germany); rabbit anti-Nanog (D73G4) and rabbit anti-Sox2 (D6D9) in 1:1000 dilution were from Cell Signaling Technology (Beverly, MA, USA). Secondary antibodies horse-radish peroxidase (HRP)-conjugated anti-rabbit and HRP-conjugated anti-mouse from Cell Signaling Technology (Beverly, MA, USA) were used in 1:4000 dilution. The immunoblots were developed using the Clarity Western ECL Substrate Bio-Rad (Hercules, CA, USA), detected with the ChemiDoc MP System (Bio-Rad), and then analyzed using Image Lab 4.0 software (Bio-Rad). Densitometric analysis of the membranes was done similarly as described elsewhere [29]. Briefly, three separate Western blots performed for each protein were analyzed. The band’s volume intensity (the luminescence signal’s intensity in the whole volume of the detected protein band—without overexposure) was measured. The bands were standardized to signal from the whole protein content in the analyzed lane (Ponceau S).

### 2.8. Cell Imaging with Atomic Force Microscopy (AFM)

AFM imaging was performed using MFP 3D-BIO AFM (Asylum Research, Oxford Instruments) and utilizing cantilever OMCL TR400PSA HW (Olympus). Cells were cultured on glass coverslips (ϕ 24) for 24 h, then fixed with 4% formaldehyde in PBS and dried. Imaging was done in contact mode in the air. The scanning area was set to 90 µm, set point to 1 V, integral gain to 10, proportion gain to 0, scan rate 1 Hz, and 1024 scan points. Gwyddion software was used to analyze the obtained images.

### 2.9. Single-Cell Force Spectroscopy (SCFS)

SCFS measurements were performed using a NanoWizard II AFM (JPK Instruments, Berlin, Germany) mounted on top of an Axiovert 200 inverted microscope (Carl Zeiss, Jena, Germany). For the cell mechanical properties measurements, standard pyramidal tipped cantilevers OMCLTR 400PSA HW (Olympus) had a nominal spring constant of 0.02 N/m. The cantilevers were calibrated by the thermal noise method before each experiment [30]. Cells were cultured on glass coverslips (ϕ 24 mm) for 24 h before the experiment. All experiments were performed at 37 °C using a temperature-controlled BioCell chamber JPK Instruments (Berlin, Germany). Force-distance curves were collected with a force load of 0.4 nN and at a rate of 2.5 μm/s. Measurements were always performed over the nuclear region of the cells. Five curves were acquired for every cell, and in every experiment, a minimum of 30 cells was analyzed. Force-distance curves were analyzed with JPK data processing software. Cell mechanical properties were acquired by evaluating Young’s modulus (E) of the cell, applying the Hertz-Sneddon model [31].

### 2.10. BrdU Cell Proliferation Assay

The BrdU Cell Proliferation Assay Kit detects 5-bromo-2-deoxyuridine (BrdU), a pyrimidine analog incorporated into the newly synthesized DNA of proliferating cells in place of thymidine. Detection of proliferating cells is based on primary antibodies that recognize BrdU, HRP-linked secondary antibodies, and TMB (HRP substrate). To perform cell proliferation assay, 6000, 3000, and 1500 scr and sh-Tβ4 cells were seeded into wells of 96-well plates 24, 48, and 72 h before the experiment, respectively. In comparing two cell lines, WM1341D and A375, 1500 cells were seeded for every time point. Estimation of cell proliferation rate was performed using the BrdU Cell Proliferation Assay Kit BioVision Inc. (South Milpitas Blvd., CA, USA) according to the manufacturer’s instructions. Signal was measured at 450 nm using a microplate reader µQuant (BioTek Instruments Inc., Bad Friedrichshall, Germany).

### 2.11. Cell Cycle Analysis

The cells growing in complete medium for 24, 48, and 72 h in 6-wells plates were harvested by trypsinization and then washed with PBS. Cells were fixed by incubation in 70% ethanol overnight at −20 °C. Next, the cells were washed three times with ice-cold PBS (5 min at 1000× *g*). After the last centrifugation, the cells were resuspended in 10 μg/mL RNAse A solution and incubated for 45 min at room temperature (RT). Subsequently, cells were incubated in 50 µg/mL propidium iodide for 30 min at RT in the dark. Samples were transferred onto the ice before measurements. The cell cycle profile was determined using the Novocyte flow cytometer (ACEA) and the ACEA NovoExpress software (ver. 1.2.4, ACEA Biosciences). At least 10,000 single gated cells were analyzed for each sample.

### 2.12. Statistical Analysis

All data are given as means ± standard deviations (SD) or standard of the mean (SEM), average-max-min, and represent at least three independent experiments. Their significance was determined using either the two-tailed, unpaired Student’s t-test or ANOVA (one-way or two-way) with posthoc Tukey HSD were applicable. Statistical analyses were performed with the help of GraphPad Prism 7 or 8 software from GraphPad Software Inc. (San Diego, CA, USA). The significance level was set at *p* < 0.05 (*), *p* < 0.01 (**), *p* < 0.001 (***) or *p* < 0.0001 (****). Graphs were plotted in GraphPad Prism 7 or 8.

## 3. Results

### 3.1. Some A375 Clones with Low TMSB4X Expression Exhibited an Impaired Ability to Form Colonies in Soft Agar

In our previous research, we described the role of Thymosin β4 in melanoma cells’ adhesion, influencing their invasion and migration abilities. To analyze the functions of Tβ4, we examined melanoma cell lines differing in the level of expression of this polypeptide. Additionally, we obtained stable clones with silenced *TMSB4X* expression using the RNA interference technique (sh-Tβ4 clones). Here, we continued the study on the role of Tβ4 in melanoma tumorigenesis using, as previously, the two melanoma cell lines: WM1341D, which is characterized by a low expression of *TMSB4X*, and A375, which was shown to have a higher expression level of *TMSB4X* [8]. We also investigated five stable clones of A375 cells with silenced *TMSB4X* expression (sh-Tβ4 clones) and finally scrambled control cells (scr). An exhaustive characterization of the A375 stable clones can be found in the paper from Makowiecka et al. [8]. There is shown the specific *TMSB4X* silencing effect of shRNAs used to obtain the stable clones.

**Figure 1:**
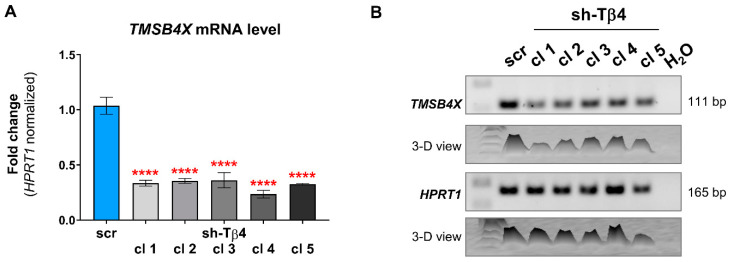
Estimation of efficiency of silencing of *TMSB4X* expression in A375 cells. Stable clones with lowered *TMSB4X* expression were obtained using shRNA constructs. (**A**) qPCR analysis of *TMSB4X* expression level. As templates, cDNAs of the A375 scr and sh-Tβ4 clones served. The results were normalized against the *HPRT1* gene and scr cells (*n* = 3). The significance level was set at **** *p* < 0.0001. The graph indicates means ± SD. (**B**) RT-PCR analysis of *TMSB4X* expression level. 2% Tris-acetate-EDTA (TAE)-agarose gel was used to separate DNA fragments.

For the sake of this study, we extracted RNA again from the sh-Tβ4 clones and transcribed it into cDNA, which served as a template in qPCR and RT-PCR reactions. qPCR and RT-PCR analyses showed that A375 stable clones retained previously reported the reduction of *TMSB4X* expression (Figure 1A,B). As discussed in our previous paper, we could not assess the expression of *TMSB4X* at the protein level, as two antibodies tested by us recognize as well Tβ10 and Tβ15 [8].

**Figure 2:**
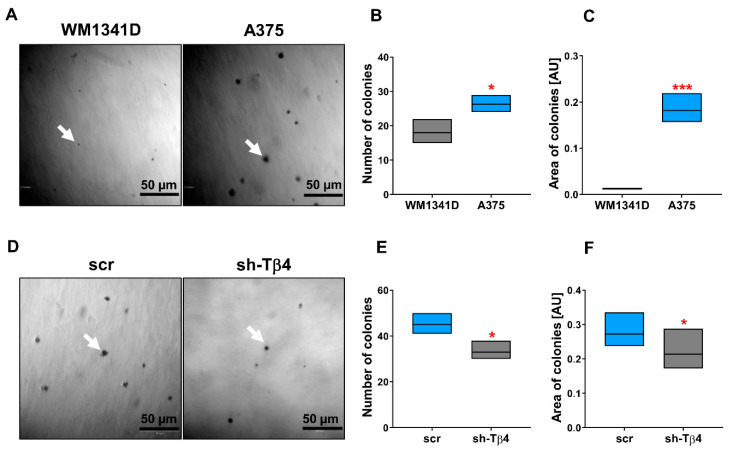
Characterization of WM1341D (low *TMSB4X* expression level) and A375 (high *TMSB4X* expression level) cells’ colony formation abilities in the soft agar colony formation assay and the effect of *TMSB4X* expression silencing on this parameter. (**A**) Representative phase-contrast images of colonies. White arrows indicate a single colony. (**B**) Quantitative analysis of the number of colonies (*n* = 3^). (**C**) Analysis of colonies’ area (*n* = 3^). (**D**–**F**) Analysis of colony formation ability of A375 control and sh-Tβ4 cells. (**D**) Representative phase-contrast images of colonies formed by the A375 clones. White arrow point at a single colony. (**E**) Quantitative analysis of colonies (*n* = 3^). (**F**) Analysis of colonies’ area (*n* = 3^). Measurements were performed with ImageJ software, which expressed results in arbitrary units (AU). The significance level was set at * *p* < 0.05, *** *p* < 0.001. Graphs indicate average-max-min values. ^The number of colonies formed and their area was calculated based on 21 microscopic pictures for every condition obtained from three separate experiments. The results presented separately for every clone are shown in Supplementary Figure S1.

We started our studies by analyzing the cells’ anchorage-independent growth ability by performing a soft agar colony formation assay. It has been shown that cell adhesion molecules play a role in stemness, as reviewed by Farahani et al. [32]. Under the test conditions, only cells exhibiting stem cell features can initiate a colony [26]. We observed that a higher number of created colonies characterized A375 cells, on average 25, compared to WM1341D cells, which formed an average of 18 colonies (Figure 2A,B). Moreover, colonies formed by A375 cells were ~15-times larger than those of WM1341D cells (Figure 2C). The tiny size of potential WM1341D cell colonies could suggest that these are the remains of initially seeded cells. In the cells with a lowered level of Tβ4, we observed diminished abilities to form colonies in comparison to control cells (Figure 2D–F). The average number of colonies was 36 for sh-Tβ4 cells and 45 for scr cells, and the area of colonies formed by sh-Tβ4 cells was smaller by about 15% comparing to scr cells (Figure 2F). The cells with a higher level of Tβ4 were characterized by an increased ability to initiate colony formation, which were more prominent in size (Figure 2D–F). Though data presented in Figure 2D–F show that Tβ4 level lowering affects the number and area of colonies, analysis of these parameters for separate clones point at diverse response of the clones to diminished *TMSB4X* expression (Supplementary Figure S1A,B).

Altogether, these results imply that A375 cells can form colonies in soft agar. However, only in a part of A375 cells silencing of *TMSB4X* expression leads to alterations in these cells’ ability to form colonies in soft agar.

### 3.2. Part of A375 Melanoma Cells with Lowered Tβ4 Level Had Diminished Tumorigenic Potential

**Figure 3:**
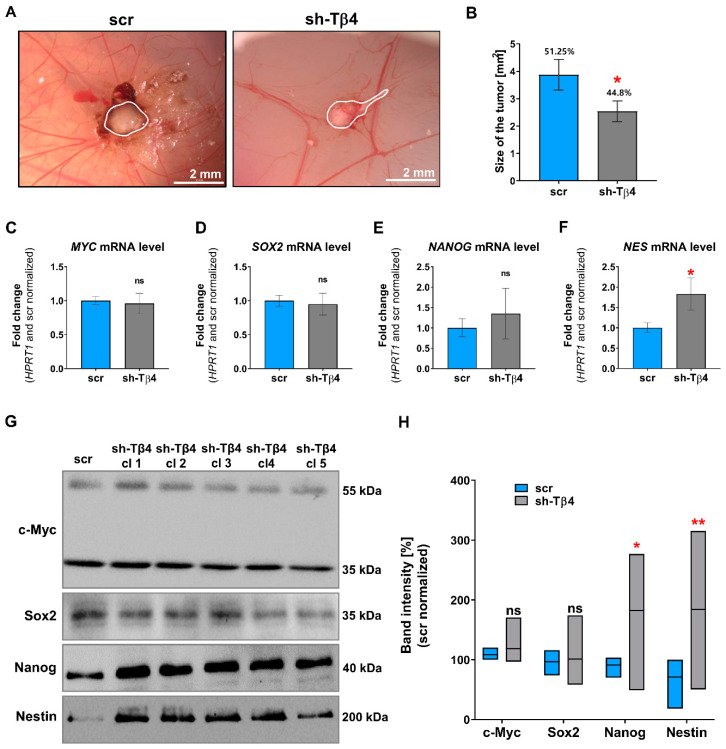
Evaluation of tumorigenic potential and level of stemness markers for the cells with lowered Tβ4 level. (**A**,**B**) The chorioallantoic membrane (CAM) model was used to estimate in vivo tumor formation by scr and sh-Tβ4 cells. (**A**) Representative microscopic images. Tumors were outlined with a white line. (**B**) Quantitative analysis of tumors and their size. For the sake of the estimation of cells’ ability to form tumors, we engrafted the cells on 33 and 38 embryos in the case of scr and sh-Tβ4 cells, respectively. To evaluate the size of tumors, 17 tumors for both types of clones were measured. The graph indicates the mean ± SEM. The results presented separately for every clone are shown in Supplementary Figure S2. (**C**–**H**). Analysis of the expression of stemness-related markers (c-Myc, Sox2, Nanog, Nestin) in scr and shTβ4 cells (*n* = 3). (**C**–**F**) qPCR analysis of *MYC*, *SOX2*, *NANOG*, and *NES* expression (*n* = 3). The *HPRT1* gene was used for the normalization. The graphs indicate the mean ± SD. The results presented separately for every clone are shown in Supplementary Figure S3. (**G**) Representative immunoblots of tested stemness-related markers. Thirty micrograms of protein were loaded on every lane. Membranes were probed for chosen stemness marker proteins: c-Myc, Sox2, Nanog, and Nestin. Corresponding Ponceau S membrane stainings are shown in Supplementary Figure S4A. (**H**) Densitometric analysis of c-Myc, Sox2, Nanog, and Nestin Western blots (*n* = 3). The significance level was set at * *p* < 0.05, ** *p* < 0.01. Graphs indicate average-max-min values. The results presented separately for every clone are shown in Supplementary Figure S4B.

To further validate the tumorigenic potential of melanoma cells with silenced *TMSB4X* expression, we decided to check in vivo tumor formation using the chorioallantoic membrane (CAM) assay often used for such purposes [33]. shTβ4 cells showed low tumor formation potential compared to control cells. We observed tumor formation in 44.8% of chicken embryos upon engraftment of sh-Tβ4 cells, while for control cells, we observed tumors in 51.25% of embryos (Figure 3A,B). The tumors formed by the cells with lowered Tβ4 levels were about 35% smaller than those observed in embryos after engraftment of scr cells (Figure 3A,B). However, when the clones were analyzed separately, we noticed no statistically significant differences for any clone than control cells (Supplementary Figure S2).

Next, in studied cells, we checked the mRNA level of transcripts coding for selected proteins implicated in stemness features, such as Nanog, Nestin, c-Myc, and Sox2 [34]. The qPCR analysis indicated statistically significant differences only in Nestin expression (Figure 3C–F and Supplementary Figure S3). Here, increased *NES* expression was noted in the case of every sh-Tβ4 clone. Next, we performed Western blot analysis of Nanog, Nestin, c-Myc, and Sox2. We carried out total protein analysis to control the amount of loaded protein on the lanes (Supplementary Figure S4A). The reason for not using standard loading controls, such as actin or tubulin, is explained elsewhere [35]. Briefly, it was shown that neither β actin nor β tubulin is suitable loading controls [36,37]. We observed similar c-Myc and Sox2 levels for both sh-Tβ4 and scr cells (Figure 3G,H). Here, it is crucial to note that we detected two forms of c-Myc, one around 35 kDa and the other of 55 kDa, both of them are correct, as two full-length forms of c-Myc protein were described (non-AUG and AUG-initiated form) [38], along with the detection of c-Myc short (35 kDa) (c-Myc S) [39]. Surprisingly, compared to control cells, we detected increased Nestin and Nanog levels in the cells with silenced *TMSB4X* expression. For sh-Tβ4 clones, we observed a 2.5-fold and 2-fold increase in the Nestin and Nanog level, respectively, relative to the control cells (Figure 3H). Yet, only for a part of the clones were statistically significant increases in the Nanog and Nestin level (Supplementary Figure S4B). We also analyzed the level of these proteins in A375 and WM1341D cells. In A375 cells characterized by better colony formation abilities, we observed a higher level of stemness marker proteins: Sox2, Nestin, and Nanog than in WM1341D cells (Supplementary Figure S5). For c-Myc, we did not observe any differences in its level between A375 and WM1341D cells.

It is important to note that Nestin’s signal in non-manipulated A375 cells was quite a week (exposure time of the membrane was 140 s) (Supplementary Figure S5A). While in the case of WB of A375 clones, we have indeed a very week signal for scr cells and quite strong for sh-Tβ4 clones (exposure time of the presented membrane was 60 s) (Figure 3G). We had to take a shorter exposure time while analyzing the A375 clones because otherwise, the bands for sh-Tβ4 would be overexposed, which would exclude them from the densitometric analysis. However, to show that the Nestin level in A375 and scr cells is similar, we analyzed the lysates of A375 and WM1341D cells and scr and sh-Tβ4 cells on one membrane, which was next incubated with antibodies recognizing Nestin (Supplementary Figure S6).

Lowering the Tβ4 level in melanoma cells gave equivocal information about the role of Tβ4 in A375 tumor formation abilities. Moreover, at the same time, we did not observe for all clones changes in the expression level of transcription factors involved in stemness. Albeit, we can state that Nestin was upregulated in almost all clones with the silenced expression of *TMSB4X*.

### 3.3. Lowering of TMSB4X Expression Did Not Affect Cell Proliferation Rate of A375 Cells

**Figure 4:**
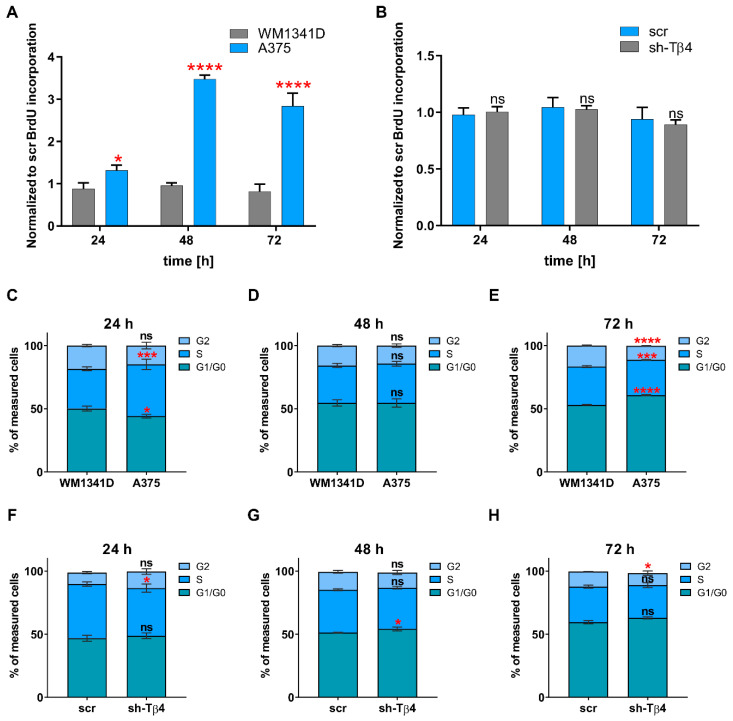
Analysis of cell proliferation rate and cell cycle progression in tested melanoma cells and evaluating the effect of *TMSB4X* expression silencing on these parameters. (**A**,**B**) Cell proliferation was measured using the BrdU assay 24, 48, and 72 h after seeding the cells (*n* = 3): (**A**) WM1341D and A375 cells (**B**) scr and sh-Tβ4 clones. The graphs indicate the mean ± SD. The results presented separately for every clone are shown in Supplementary Figure S7. (**C**–**H**) Flow cytometric analysis of cell cycle with propidium iodide DNA staining (*n* = 3). (**C**–**E**) Analysis of the cell cycle was performed on WM1341D, and A375 cells collected, respectively, 24, 48, and 72 h after seeding. (**F**–**H**) Analysis of the cell cycle was performed on scr and sh-Tβ4 cells collected 24, 48, and 72 h after seeding, respectively. The graphs indicate the mean ± SD. The results presented separately for every clone are shown in Supplementary Figure S8. The significance level was set at * *p* < 0.05, *** *p* < 0.001, and **** *p* < 0.0001.

Next, we evaluated cell proliferation rate in tested melanoma cell lines and stable clones using the BrdU cell proliferation assay basing on the incorporation of thymidine analog during DNA synthesis [40]. We decided to do it because Huang and colleagues showed that Tβ4 influenced HL-60 cell proliferation [41]. We observed a significant difference in the proliferation rate when comparing WM1341D and A375 cells for 24, 48, and 72 h after subculturing (Figure 4A). In A375 cells, the proliferation rate was higher by about 1.5, 3.6, and 3.5 times for 24, 48, and 72 h, respectively, compared to WM1341D cells. On the contrary, we did not detect any significant difference in the proliferation rates between sh-Tβ4 and scr cells (Figure 4B). Additionally, the analysis performed for every sh-Tβ4 clone separately corroborated this observation (Supplementary Figure S7).

Next, we decided to analyze the cell cycle in the tested cells, as Tβ4 impacted cyclin b1 level in HeLa cells [42]. The method used was based on the fluorescent labeling of the cells’ genetic material with propidium iodide, followed by the cells’ analysis with flow cytometry [43]. The resulting histogram of fluorescence intensity consists of three cell populations: G1/G0 with a single copy of DNA, G2 with doubled DNA content, and S phase. The fluorescent intensity signal is between values obtained for G1/G0 and G2 phase, for which analysis of cell cycle graphs for A375 and WM141D cells collected 24, 48, and 72 h after cell seeding indicated some changes in cell distribution between phases (Figure 4C–E). After 24 h, around 10% more A375 cells were in the S phase, and about 6% fewer cells in G1/G0 than observed for WM1341D cells (Figure 4C). The most significant differences were detected in tested melanoma cell lines collected 72 h after cell seeding (Figure 4E). In that case, about 13% more A375 cells were in G1/G0 phase and simultaneously around 5% fewer cells were in G2 phase compared to WM1341D cells. Similar to the BrdU cell proliferation assay, the cell cycle analysis did not reveal any significant differences in distribution of the cells with downregulated expression of Tβ4 between phases (Figure 4F–H and Supplementary Figure S8).

In summary, we can state that there are no significant differences in the proliferation rate and cell cycle after silencing *TMSB4X* expression in A375 cells.

### 3.4. Lower Tβ4 Level Was Correlated with Alterations in Actin Cytoskeleton and Intermediate Filaments Organization

We also examined the cellular localization of some components of the cytoskeleton. We focused on filamentous (F-) and monomeric (G-) actin because of the role of Tβ4 in the actin cytoskeleton organization [1]. We also checked Nestin’s subcellular localization as it is the intermediate filament protein of neuronal stem cells [44], and we observed its increased level in sh-Tβ4 cells (Figure 3G,H). Additionally, we looked at Vimentin because it has been shown that this intermediate filament protein is a Nestin polymerization partner [45]. In previous work, we observed the lowered Vimentin level in the cells with silenced expression of *TMSB4X* [8].

**Figure 5:**
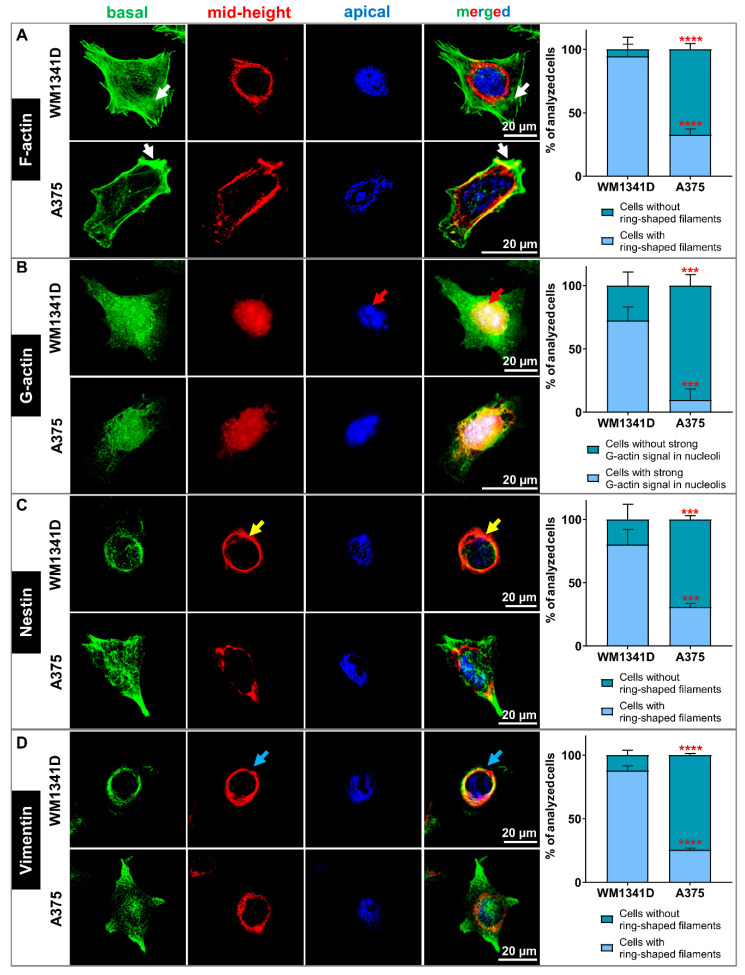
The actin cytoskeleton and organization of Nestin and Vimentin filaments in WM1341D and A375 cells. (**A**–**D**) Immunocytochemical stainings to detect selected cytoskeleton components in WM1341D and A375 cells. The confocal microphotographs pictures for each cell were captured at three focal planes: cell contact area to the substratum (basal—green), a cross-section of the cell nucleus (mid-height—red), and nucleus apical outer surface (apical—blue). Finally, the photos were merged into a single image. (**A**) Detection of filamentous actin (F-actin). White arrows indicate stress fibers. (**B**) Visualization of monomeric actin (G-actin). Red arrows point at the denser aggregation of G-actin in nucleoli. (**C**) Detection of subcellular localization of Nestin. Yellow arrows indicate a ring-shaped localization around the nucleus. (**D**) Immunocytochemical staining to detect Vimentin. Blue arrows indicate a ring-shaped structure around the nucleus. Separated microphotographs with additional F-actin and cell nucleus visualization are presented in Supplementary Figures S9A, S11A and S13A for G-actin Nestin- and Vimentin-staining, respectively. In the case of Supplementary Figures S10A, S12A, and S14A, population photos are presented. We quantified our observations and presented the data in the form of graphs indicating the mean ± SD. At least 30 cells from 3 population photos per condition were analyzed. The significance level was set at *** *p* < 0.001, and **** *p* < 0.0001.

We detected F- and G-actin using fluorescently labeled phalloidin and deoxyribonuclease I (DNase), respectively. The intermediate filament proteins were detected with the help of appropriate antibodies. The confocal microphotographs for each cell were taken in three focal planes: cell contact area to the substratum (basal—green), a cross-section at the level of the cell nucleus (mid-height—red), and nucleus apical outer surface (apical—blue) (Figure 5). Finally, the photos taken at different focal planes were merged into a single image. Such a presentation of a given protein’s subcellular localization has already been reported by others [46]. Thanks to this approach, we indicated the 3D subcellular localization of the studied proteins. Simultaneously, we visualized F-actin and cell nucleus at every analyzed focal plane. Microphotographs are shown in Supplementary Figures S9A, S11A and S13A for G-actin-, Nestin- and Vimentin-staining, respectively. In the case of Supplementary Figures S10A, S12A, and S14A, population photos are presented.

Figure 5A and Supplementary Figures S9A and S10A present the organization of filamentous actin in tested melanoma cell lines. We observed more pronounced F-actin structures at the basal focal plane (green) in WM1341D cells, including stress fibers (pointed by arrows) than for A375 cells. At the mid-height plane (red), we observed more polymerized actin around the cell’s nucleus, forming a ring for WM1341D cells than for A375 cells. Finally, in the apical part of the WM1341D cells (blue), the actin network was more compact than in A375 cells. In both WM1341D and A375 cells, the G-actin was localized in the cytoplasm and the cell nucleus (Figure 5B and Supplementary Figures S9A and S10A). However, in WM1341D cells, a robust signal for G-actin was observed in nucleoli (Figure 5B—red arrows).

Nestin’s visualization in WM1341D cells showed an interesting pattern. This intermediate filament protein was localized predominately in the nuclear area, forming a structure entwining the cell nucleus (Figure 5C and Supplementary Figures S11A and S12A). At the mid-height plane (red), Nestin filaments were assembled in the ring-shaped form (Figure 5C—yellow arrows). We observed Nestin filaments below and above the nucleus in the other two focal planes. On the contrary, in A375 cells, Nestin was primarily localized in the basal area’s cell body. The observed Vimentin staining pattern was similar to that described previously for Nestin for both cell lines (Figure 5D and Supplementary Figures S13A and S14A).

**Figure 6:**
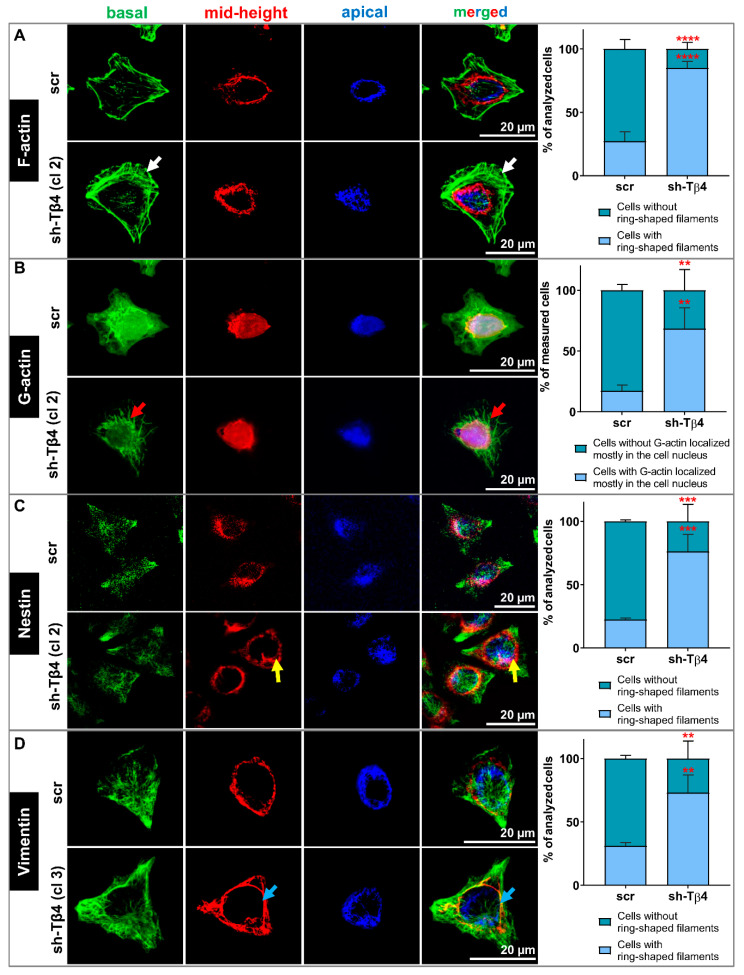
The role of Tβ4 in the actin cytoskeleton and organization of Nestin and Vimentin filaments in scr and sh-Tβ4 cells. (**A**–**D**) Immunocytochemical stainings to detect selected cytoskeleton components in scr and shTβ4 cells. The confocal microphotographs pictures for each cell were captured at three focal planes: cell contact area to the substratum (basal—green), a cross-section of the cell nucleus (mid-height—red), and nucleus apical outer surface (apical—blue). Finally, the photos were merged into a single image. (**A**) Detection of filamentous actin (F-actin). White arrows indicate stress fibers. (**B**) Visualization of monomeric actin (G-actin). Red arrows indicate the denser aggregation of G-actin in the cytoplasm. (**C**) Detection of subcellular localization of Nestin. Yellow arrows indicate a ring-shaped localization around the nucleus. (**D**) Immunocytochemical staining to detect Vimentin. Blue arrows indicate a ring-shaped structure around the nucleus. Separated microphotographs with additional visualization of F-actin and cell nucleus are presented in Supplementary Figures S9B-B’’ and S10B-B’; S11B-B’’ and S12B-B’; S13B-B’’ and S14B-B’ for G-actin, Nestin- and Vimentin-staining, respectively. In the case of Supplementary Figures S10B–B’, S12B-B’, and S14B-B’ population photos are presented. We quantified our observations and presented the data in the form of graphs indicating the mean ± SD. At least 30 cells from 3 population photos per condition were analyzed. The significance level was set at ** *p* < 0.01, *** *p* < 0.001, and **** *p* < 0.0001.

Next, we looked at the subcellular location of F-actin, G-actin, Nestin, and Vimentin in scr and sh-Tβ4 cells. In the case of sh-Tβ4 cells, we noted more pronounced F-actin structures at the basal focal plane (green) when compared to scr cells (Figure 6A, Supplementary Figures S9B and S10B). Similarly to WM1341D, we noticed more polymerized actin around the cell’s nucleus for sh-Tβ4 than for control cells at the mid-height plane. At the apical plane, less compact F-actin was observed for scr cells than for sh-Tβ4 cells. In the cells with downregulated *TMSB4X* expression, G-actin’s signal was weaker than in control cells and localized mainly in the cell nucleus (Figure 6B, Supplementary Figures S9B and S10B). In sh-Tβ4 cells, freely dispersed G-actin was absent from the cytoplasm. In sh-Tβ4 cells, more Nestin filaments were localized around the cell nucleus at the mid-height and apical planes than in control cells (Figure 6C, Supplementary Figures S11B and S12B). We observed a similar Nestin staining pattern for Vimentin in tested cells (Figure 6D, Supplementary Figures S13B and S14B). Additional merged focal planes for every staining for clones not shown in Figure 6 are shown in Supplementary Figures S9B’, S11B’, and S13B’. Corresponding panels presenting all stainings separately for every clone are shown in Supplementary Figures S9B’’, S11B’’, and S13B’’. Finally, accompanying population photos of these clones are presented in Figures S10B’, S12B’, and S14B’.

In summary, the actin cytoskeleton, Nestin, and Vimentin filaments were organized significantly differently in melanoma cells with low Tβ4 levels than the cells with high *TMSB4X* expression. Less prominent F-actin structures were observed for latter cells, accompanied by Nestin and Vimentin being more dispersed at the basal focal plane. It is important to note here that these observations were made for all sh-Tβ4 clones.

### 3.5. Tβ4 Influenced the Height of the Melanoma Cells

**Figure 7:**
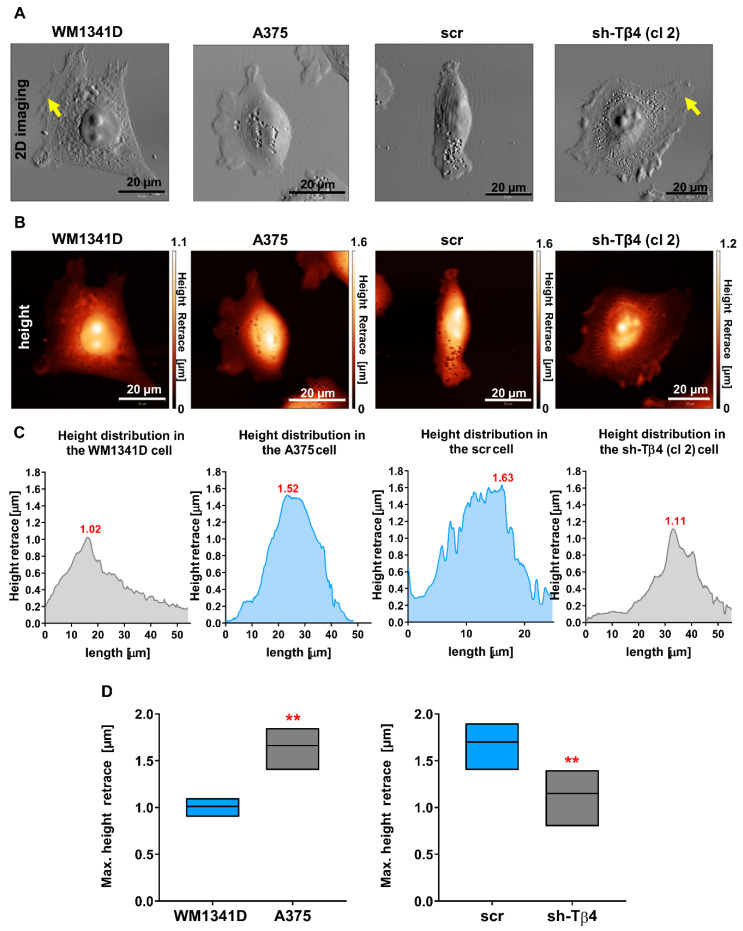
Tβ4 influences melanoma cell morphology. (**A**) 2D visualization as a grayscale map of the cell surface of tested melanoma cells using the atomic force microscopy (AFM). Yellow arrows point at filamentous structures. (**B**) WM1341D, A375, scr, and sh-Tβ4 cells’ body height measurements by AFM. The data obtained from the height measurements were visualized by a heat map (height retrace) of the cell body. The black color corresponds to 0 μm, passes through various red and yellow shades, and the highest point of the tested object is marked with white. (**C**) Histograms present the difference in the height of the cells shown in A-B in correlation to a drawn line’s position across the cell. (**D**) Graphs are illustrating the maximal height of the cells (*n* = 5–10). Graphs indicate average-max-min values. The significance level was set at ** *p* < 0.01. The results presented separately for every clone are shown in Supplementary Figure S15D.

We performed 2D imaging, acquiring the *error* signal, which provides highly contrasted lateral resolution, and 3D imaging attaining the actual height of tested melanoma cells using an atomic force microscope. 2D Topography images of WM1341D and sh-Tβ4 cells were similar (Figure 7A and Supplementary Figure S15A). These cells were more spread on the substrate than A375 and scr cells. Moreover, WM1341D and sh-Tβ4 cells were characterized by broader membrane extensions in which we observed more prominent filamentous structures (Figure 7A and Supplementary Figure S15A—pointed with arrows) compared to A375 and scr cells, respectively. In WM1341D and sh-Tβ4 cells, the nucleus was distinctly separated from the rest of the cell body with clearly visible nucleoli in contrast to A375 and scr cells. The maximum heights of the presented A375 and scr cells were 1.5 µm and 1.6 µm, respectively. While in the case of WM1341D and sh-Tβ4 clone no. 2 cells, they were 1.02 µm and 1.11 µm high, respectively (Figure 7B,C). In these cells, the highest points in the cell body were nucleoli. Body heights for other sh-Tβ4 clones are presented in Supplementary Figure S15B,C. There were observed statistically significant differences in the body heights, i.e., A375 and scr cells were much higher than WM1341D and sh-Tβ4 cells (Figure 7D and Supplementary Figure S15D).

In summary, melanoma cells with low Tβ4 levels were characterized by a more distinct morphology and lesser height of the cell body than the cells with a high level of this polypeptide. This phenomenon was observed for all sh-Tβ4 clones.

### 3.6. Lower Level of Tβ4 Was Positively Correlated with Cells Being Stiffer

**Figure 8:**
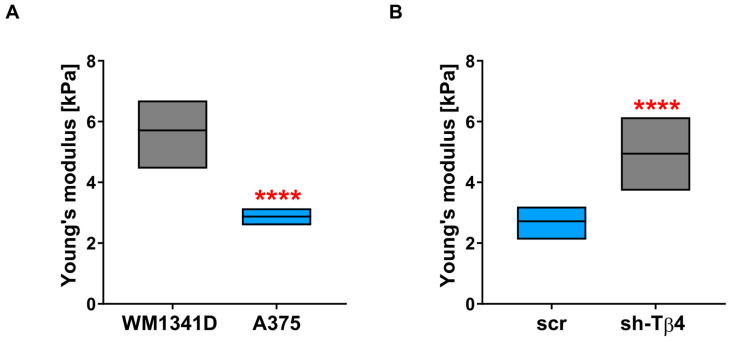
Analysis of the role of Tβ4 in the mechanical properties of melanoma cells. Single-cell force spectroscopy (SCFS) measurements were performed to calculate Young’s modulus for tested melanoma cells. For every cell, five curves were acquired. The average values of Young’s modulus are presented in the graphs: (**A**) for WM1341D and A375 cells (*n* = 30) and (**B**) for scr (*n* = 30) and sh-Tβ4 clones (*n* = 30 for every clone). The significance level was set at **** *p* < 0.0001. Graphs indicate average-max-min values. The results presented separately for every clone are shown in Supplementary Figure S16.

Finally, we performed an analysis of the tested melanoma cells’ mechanical properties using single-cell force spectroscopy (SCFS). Measurements were conducted over the nuclear region of the cells. We could have decided to perform experiments at the cell periphery. However, according to the literature, the outcomes obtained for that area of a cell could be adulterated by the proximity of the cantilever tip to the substrate on which cells grow [47,48]. That’s why we have chosen the nuclear region of the cells to perform this analysis. Obtained force-distance curves were next analyzed with the appropriate AFM software to calculate for each cell Young’s modulus defining cell stiffness. WM1341D cells were characterized by higher values of Young’s modulus, which means that these cells were stiffer than A375 cells (Figure 8A). The average value of Young’s modulus for WM1341D cells was 5.6 kPa, almost 2 times higher than for A375 cells, for which the average value was 2.8 kPa. The stiffness analysis also showed that the stiffness was increased for the cells with decreased *TMSB4X* expression (Figure 8B, Supplementary Figure S16). The average value of Young’s modulus for sh-Tβ4 cells was around 5 kPa and was almost twice that of the control cells, for which the average value was ~2.7 kPa.

Our results suggest that the silencing of *TMSB4X* expression in melanoma cells caused changes in their biomechanical properties. Again, this effect was observed for every sh-Tβ4 clone.

## 4. Discussion

Standard anti-cancer treatment encompassing surgical resection, chemotherapy, and radiotherapy is ineffective at eradicating cancer stem cells [10]. The presence of this cell subpopulation in the tumor is often the reason for tumor recurrence and progression. For most human cancers, the percentage of the cells capable of de novo tumor formation ranges from 0.1 to 0.0001% [49]. For this reason, studying tumor-initiating cell subpopulation is essential but it is also challenging. It has been shown that some established metastatic melanoma cell lines possess stem-cell-like subpopulations [12]. Because Wirsching et al. [9] showed that Tβ4 is essential for the maintenance of glioblastoma stemness, we decided to establish the role of Tβ4 in melanoma tumorigenicity and clonogenicity as these factors are manifestations of stemness features [24].

According to the literature, overexpression of Tβ4 was observed in several types of tumors, such as malignant renal tumors, thyroid, colon and non-small cell lung cancers [50], and melanoma [51]. Our results indicate that the cells with a lowered Tβ4 level initiated a lower number of CAM model tumors than control cells. Moreover, they were smaller. The clonogenic potential was as well diminished in those cells. Currently, approximately 25 transcription factors have been characterized to be active in stem cells [52]. Analysis of anchorage-independent growth leading to melanoma colonies’ formation (melanophores) indicated a very heterogeneous stemness marker expression [53]. We decided to analyze the levels of three transcription factors: c-Myc, Nanog, and Sox2. Expression of Myc proto-oncogene protein (c-Myc) has been connected with tumorigenesis in mouse models and observed in up to 70% of human cancers [54]. It has been shown that the expression of Nanog is involved in the regulation of the epithelial-mesenchymal transition (EMT) and chemoresistance in ovarian cancer [55]. Sox2, on the other hand, is expressed in multipotential neural stem cells and is essential to maintain their proliferative potential [56]. Additionally, we examined the Nestin level in tested melanoma cells. This protein is a cytoskeletal intermediate filament protein initially detected in neural stem cells, for which it is essential for functioning [44]. We did not observe the unequivocal correlation between tumorigenic potential and chosen stemness-related transcription factors’ expression level in tested melanoma cells. The amount of c-Myc and Sox2 remained unaltered. Still, the Nanog level was increased in some clones upon *TMSB4X* expression silencing. It is not surprising, as Perego and colleagues did not find any direct correlation between tested stemness-related markers and tumorigenicity in melanophores [53].

**Figure 9:**
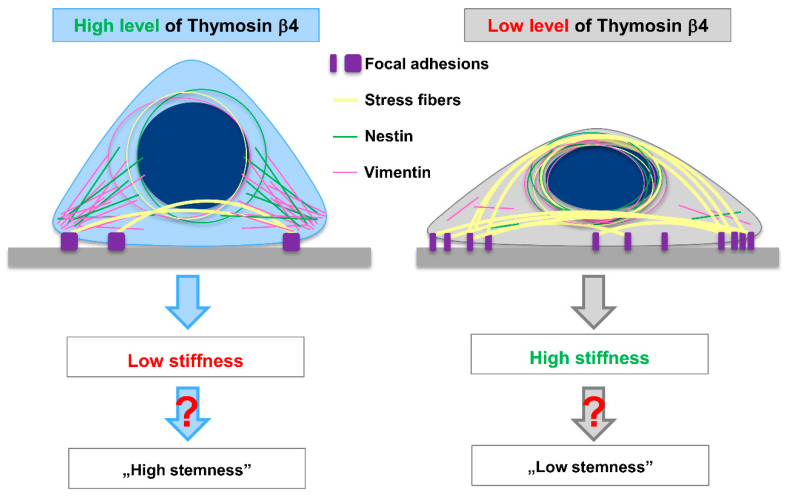
A summary of obtained results. Accordingly to the results obtained by us for the A375 cells with decreased *TMSB4X* expression, lowering Tβ4 level leads to reorganization of focal adhesion [8]. The cells form more focal adhesion (FA) sites but of smaller size. That leads to more pronounced stress fibers and Vimentin and Nestin’s relocalization towards the perinuclear area, where “rings” of these intermediate filaments (IF) proteins are observed around the cell nucleus. Finally, the cells are more spread on the substratum [8] and become stiffer. Changes in the cells’ biomechanical properties with lowered *TMSB4X* expression influence stemness features only in a part of cell clones.

Constant crosstalk between the cytoskeleton’s three main components (actin microfilaments, microtubules, and intermediate filaments, IFs) is essential for proper cell functioning. IFs play role in tissue integrity and cell-shape determination, IFs are tissue-specific proteins [57]. Our research analyzed the distribution of two IFs: Nestin and Vimentin, in cells with downregulated expression of *TMSB4X*. Nestin is a neural stem cell marker [44], while Vimentin expression is the cells’ marker, which underwent EMT [58]. Nestin, unlike most other intermediate filament proteins, is incapable of polymerizing by itself [45]. Instead, it interacts preferentially with other IF proteins (e.g., Vimentin). A growing amount of data indicates a bidirectional interplay between actin and IFs [59]. It was shown that, during mitotic division, Vimentin controls actin organization and mechanics [60]. On the other hand, contractile actin stress fibers, which contain Myosin II, are essential for the Vimentin network’s perinuclear localization [61]. When stress fibers were disassembled in the cells, Vimentin’s perinuclear localization disappeared, and instead, Vimentin was spread across the cell body. Jiu and colleagues have also demonstrated that contractile actin stress fibers are required for the Vimentin/Nestin network’s perinuclear localization. It was shown that Nestin knockout in highly metastatic breast cancer cells increased cell stiffness [62]. However, when Nestin’s level was raised by exogenous expression in the same cell line, the obtained values of Young’s modulus were still higher than in control cells. This perplexing observation could be associated with a different distribution of Nestin in the cell body. Our results are in agreement with these studies. After lowering the Tβ4 level in melanoma cells, we observed an increased number of F-actin structures, along with Nestin and Vimentin’s location around the cell nucleus. In the more invasive cell line A375, we observed a higher Nestin level and softer cell body than for WM1341D cells, in which invasion potential is low compared to A375 cells [8]. Upon the downregulation of the Tβ4 level in A375 cells, we observed an increased level of Nestin, and slightly lowered level of Vimentin [8]. However, the redistribution of Nestin to the perinuclear area probably caused increased stiffness. Apparently, Tβ4, by affecting actin cytoskeleton, also induced changes in Nestin and Vimentin location. In that way, Thymosin β4 influences cell morphology and biomechanical properties (Figure 9).

Intriguingly, it has been shown that Tβ4 can determine cell fate only through biophysical effects [63]. Several studies correlate stemness with cellular stiffness [64]. The pluripotent state is associated with intracellular and nuclear stiffness [65]. We observed the same situation during our research. Melanoma cells, which manifested more stem cell-like features, were also characterized by the softer cell body measured by single-cell force spectroscopy (SCFS). The silencing of *TMSB4X* expression caused increased stiffness in these cells. Rotsch and Radmacher showed—based on SCFS measurements—that actin filaments play a vital role in maintaining cell stiffness [66]. Recently, the involvement of IFs in this process was also described [67]. The role of actin cytoskeleton remodeling in the differentiation of mesenchymal stem cells (MSCs) seems to be well established. However, its involvement in cancer cells’ acquisition of stem cell features needs to be more explored. New studies were published, implying that RhoC—Ras homolog gene family member C, being a GTPase involved in actin cytoskeleton remodeling, is an essential factor in CSCs maintenance [68,69,70].

There is a growing interest in integrins, which are the critical components of focal adhesions. The signaling triggered by the activation of integrins is currently intensively studied in the context of tumor initiation, the plasticity of the tumor cells, and resistance to targeted therapies as reviewed elsewhere [71]. Recently, we showed that Tβ4 plays a role in the organization of focal adhesions, which provide cells the attachment to the surface and are the link between the interior of a cell and the cell’s environment through mechanosensing [72]. Xie, with colleagues, showed that the stronger interactions with the substrate lead to better spreading of a cell with accompanying reduction in its height and volume [73]. It is in agreement with our observations for A375 sh-Tβ4 cells, i.e., these cells have changed adhesion abilities and are more spread on the substrate [8], and finally have diminished body height. Changes in biomechanical properties of a cell influence several cellular processes. The impact of stress fibers dynamics anchored to the focal adhesions on the cell nucleus’s functioning is discussed elsewhere [74]. It has been shown that the mechanical properties of materials on which cells grow strongly influence their fate and functions [75]. It is then plausible that changes in focal adhesion organization and, thus, adhesion upon *TMSB4X* expression silencing could reprogramming a cell’s fate. However, we observed a correlation between biomechanical properties and stemness solely for selected clones with silenced expression of *TMSB4X* (Figure 9).

We noticed that not all clones expressed statistically significant changes compared to the control cells while analyzing the results from clonogenicity, tumorigenicity, and expression of stemness-related markers. However, for the distribution of the cytoskeletal proteins studied here and the cells’ biomechanical properties, we obtained consistent results for all tested clones with lowered *TMSB4X* expression. This intriguing observation suggests that, though Tβ4 affects the cells’ morphology and stiffness, this polypeptide’s influence on stemness has to be dependent on other factor/s, which are not uniformly present in the whole population of A375 cells. That can be linked with melanoma cells being the highly heterogenic population of cells (e.g., in terms of the expression of stemness markers) [53]. That is in accordance with other studies showing that melanoma cells are characterized by high plasticity [76]. Thus, Tβ4′s influence on the manifestation of stemness/tumorigenic features might depend on the cellular landscape of expressed proteins in a given cell. We did not decide to pool the obtained clones and then subject them to analyses done in other studies [77,78,79]. However, we show the pooled results for sh-Tβ4 clones compared with the outcomes for control cells to draw general conclusions for whole populations of analyzed cells. We believe that showing the results for single clones might be important, proving the heterogeneity of melanoma cells again.

The phenotypic heterogeneity has been reported for several cultured already for decades cell lines, e.g., MDA-MB-231 (breast cancer) [80], Jurkat and K562 (leukemia) [81], or HeLa (cervical carcinoma) [82]. For melanoma cell lines, phenotypic heterogeneity was observed as well. With colleagues [83], Snyder observed a subpopulation of NME1^low^ cells within WM9 and WM278 cell lines. NME1 is a metastasis suppressor, and the cells with a lower level of this protein were found to invade collectively better and express higher levels of genes essential for tumor aggressiveness. Analysis of the COLO829 melanoma cell line by shallow single-cell sequencing of genomic DNA showed that this cell line consists of four major groups of subclones, implying that it is evolving [84]. Murine melanoma cell line—B16F10, upon being subjected to the limiting dilution clones isolation procedure, gave rise to clones differing in metastatic abilities [85]. Finally, there are as well studies concerning the A375 cell line phenotypic heterogeneity. Pucciarelli, with colleagues, showed that exposure of cells to hypoxia enhances their heterogeneity [86]. We have recently demonstrated that A375 cells are not homogeneous in terms of expression of *ACTBL2*, a gene coding for β-actin-like protein 2, which is the seventh actin isoform [87]. We have shown by applying the RNAscope procedure that only subsets of A375 and Hs294T (another melanoma cell line) cells expressed *ACTBL2*. To sum up, subpopulations of tumor cells are found not only in melanoma tissues isolated from patients but also in cell lines.

Phenotypic heterogeneity is a manifestation of a process called noise in gene expression [88], which refers to “the measured level of variation in gene expression among cells, regardless of source, within a supposedly identical population.” Intrinsic or extrinsic factors can cause this noise. The former can be explained by, e.g., stochastic chromatin-remodeling events, while the latter may represent the local environment’s influence on a cell. The most basic explanation of noise in protein levels is noise in gene expression. This research field is gaining more and more attention recently and focuses on how fluctuations in transcription result in different phenotypes and cell behavior. Splendid work published by Bonny and colleagues [89] presents an elegant system to study phenotypic heterogeneity, i.e., Tunable Noise Rheostat (TuNR). Using it on the PC9 cell line of human lung adenocarcinoma, the researchers proved varying degrees of gene expression heterogeneity within this cell line. Another critical study employed a computational modeling approach to study epithelial-mesenchymal heterogeneity. Basing on experimental data published for prostate cancer cells by Ruscetti et al. [90], Tripathi et al. [91] proved that epithelial-mesenchymal heterogeneity could result from the noise in the portioning of biomolecules occurring during mitosis.

Noise in gene expression favors cells as the existence of several subpopulations among an isogenic population might help in its survival under stress conditions [92]. That is why it is crucial to address noise in gene expression among tumor cells, as their potential needs to evolve/adapt and escape from therapy [93,94]. For instance, according to one of the newest studies, both epithelial and mesenchymal cells should be targeted during tumor treatment [91]. That has tremendous implications for melanoma cells in the light of our previous research on A375 and WM1341D cells, referring to Tβ4′s role in migration, invasion, and adhesion. We showed that these cell lines are intermediate between epithelial and mesenchymal phenotypes [8]. That was manifested by high Vimentin, Zeb1, and SNAI1/Snail (mesenchymal markers) levels in A375 cells, which simultaneously exhibited high levels of ZO-1 (epithelial marker). Altogether, this corroborates our hypothesis formulated in this paper that the A375 cell line is heterogeneous in tumorigenic potential. Thus, stemness features as diminished clonogenicity and tumorigenicity were reported only for some of the studied here clones.

## 5. Conclusions

Based on the results presented here, we conclude that Tβ4 is involved in melanoma cell biomechanics. An increased level of that polypeptide induces actin cytoskeleton reorganization so that fewer stress fibers inside the cell body probably cause rearrangement of the Nestin and Vimentin network. Finally, it leads to changes in the A375 cell’s topography, morphology, and stiffness. Much data is postulating the involvement of Tβ4 in gaining by the cells’ stemness feature. However, the mechanism of this process remains unknown. This paper clearly shows that Tβ4 influences the biomechanical properties of melanoma cells via reorganization of cytoskeletal components. At the same time, we decouple biomechanical properties from gaining stemness features. In A375 cells stemness is probably linked with alterations in the biomechanical properties. Still, it is not a direct link, and apparently there is an additional factor linking them, which is not uniformly present in the whole population of A375 cells. Thus, we additionally prove here that the A375 cell population is characterized by phenotypic heterogeneity. Tβ4′s influence on the manifestation of stemness features might depend on the cellular landscape of expressed proteins. Altogether, we provide new knowledge about the complex crosstalk between stemness and cytoskeleton of A375 melanoma cells. We believe that a better understanding of why some cancer cells gain stemness features will help to design new anti-tumor therapies, which preferably should target all tumor cells and not chosen subpopulations. A summary of our findings is presented in Figure 9.

## Supplementary Materials

The following are available online at https://www.mdpi.com/article/10.3390/cells10040769/s1. Figure S1: Colony formation abilities in the soft agar colony formation assay shown separately for each sh-Tβ4 clone; Figure S2: An ability of scr and sh-Tβ4 cells to form tumors in CAM assay; Figure S3: qPCR analysis of *MYC*, *SOX2*, *NANOG*, *NES* shown separately for each sh-Tβ4 clone; Figure S4: Evaluation of c-Myc, Sox2, Nanog and Nestin level shown separately for each sh-Tβ4 clone; Figure S5: Evaluation of c-Myc, Sox2, Nanog and Nestin level in WM1341D and A375 cells; Figure S6: Evaluation of Nestin level in WM1341D and A375cell, and scr and sh-Tβ4 clones; Figure S7: Analysis of cell proliferation rate shown separately for each sh-Tβ4 clone; Figure S8: Analysis of cell cycle rate shown separately for each sh-Tβ4 clone; Figure S9: The role of Tβ4 in melanoma cells’ actin cytoskeleton organization; Figure S10: The role of Tβ4 in melanoma cells’ actin cytoskeleton organization (cell population analysis); Figure S11: The role of Tβ4 in Nestin’s organization in studied cells; Figure S12: The role of Tβ4 in Nestin’s organization in studied cells (cell population analysis); Figure S13: The role of Tβ4 in organization of Vimentin in melanoma cells; Figure S14: The role of Tβ4 in organization of Vimentin in melanoma cells (cell population analysis); Figure S15: Analysis of the surface of sh-Tb4 clones and their height; Figure S16: Single-cell force spectroscopy (SCFS) measurements shown separately for each sh-Tb4 clone.

## Abbreviations

AFM	atomic force microscopy
CSCs	cancer stem cells
DNase	deoxyribonuclease I
EMT	epithelial-mesenchymal transition
FA	focal adhesion
F-actin	filamentous actin
G-actin	monomeric actin
IFs	intermediate filaments
iPSCs	induced pluripotent stem cells
mESCs	mouse embryonic stem cells
MSCs	mesenchymal stem cells
SCFS	single-cell force spectroscopy
scr	control A375 cells transfected with scrambled sh-sequence
sh-Tβ4	stable A375 clones with silenced *TMSB4X* expression
Tβ4	Thymosin β4
*TMSB4X*	gene coding for Thymosin β4
