# Supplementary material for: Changes in Biomechanical Properties of A375 Cells Due to the Silencing of TMSB4X Expression Are Not Directly Correlated with Alterations in Their Stemness Features
Source: Cells. 2021 Mar 31;10(4):769. doi: 10.3390/cells10040769 (PMC8067020; doi:10.3390/cells10040769)
Supplement: Supplementary file 1 [file cells-10-00769-s001.pdf]

Supplementary Material

Changes in Biomechanical Properties of A375 Cells Due to the Silencing of *TMSB4X* Expression are not Directly Correlated with Alterations in Their Stemness Features

Aleksandra Makowiecka [1,2]*, Ewa Mazurkiewicz [1], Ewa Mrówczyńska [1], Natalia Malek [1], Alice Battistella [2], Marco Lazzarino [2], Dorota Nowak [1] and Antonina Joanna Mazur [1]*

[1] Department of Cell Pathology, Faculty of Biotechnology, University of Wroclaw, Wrocław 50-383, Poland; aleksandra.makowiecka@uwr.edu.pl; ewa.mazurkiewicz@uwr.edu.pl; ewa.mrowczynska@uwr.edu.pl en.malek@gmail.com; dorota.nowak@uwr.edu.pl; antonina.mazur@uwr.edu.pl
[2] Istituto Officina dei Materiali-National Research Council, Trieste I-34149, Italy; lazzarino@iom.cnr.it; battistella@iom.cnr.it

*Correspondence: aleksandra.makowiecka@uwr.edu.pl (a.makowiecka1@gmail.com), antonina.mazur@uwr.edu.pl; Tel.: +48-71-375-6206 (A.J.M.)

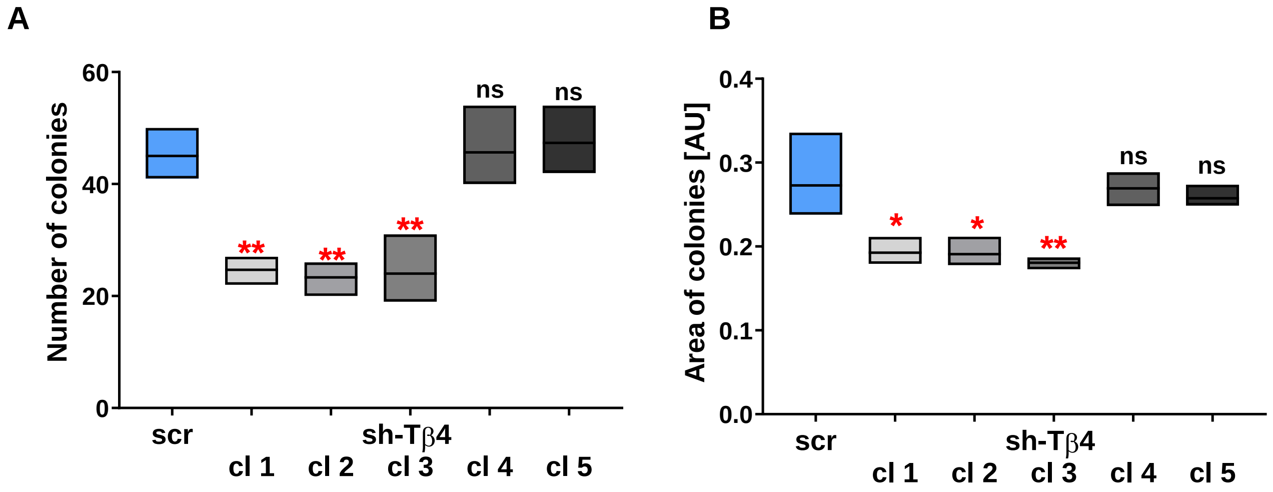

Figure S1. Colony formation abilities in the soft agar colony formation assay shown separately for each sh-Tβ4 clone. (**A**) Quantitative analysis of colonies (n = 3). (**B**) Analysis of colonies' diameter (n = 3). Graphs indicate average-max-min values. AU is an arbitrary unit from the ImageJ software. The significance level was set at *P < 0.05 and **P < 0.01. This figure corresponds to the Figure 2E-F in the main text.

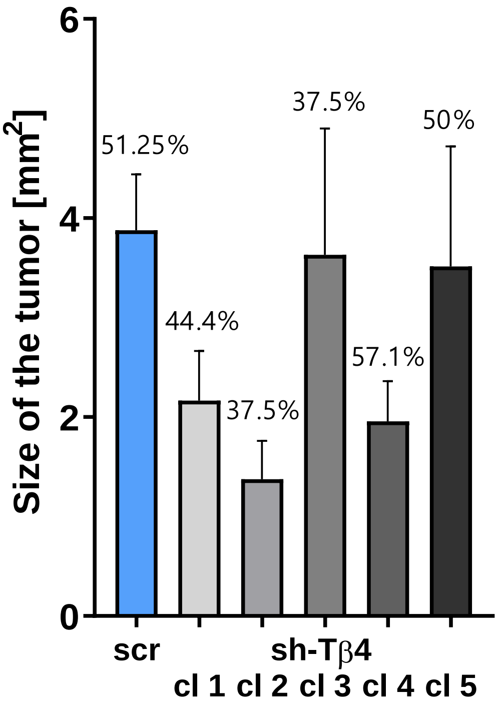

Figure S2. An ability of scr and sh-Tβ4 cells to form tumors in CAM assay. Quantitative analysis of tumors generated on chorioallantoic membrane of 7 days old chicken embryos within 7 days. The percent of formed tumors and their size were analyzed [n = 3 (for every clone) – 17 (for scr cells)]. The graph presents the mean ± SEM of formed tumors' size. The significance level was set at *P < 0.05. This figure corresponds to the Figure 3B in the main text.

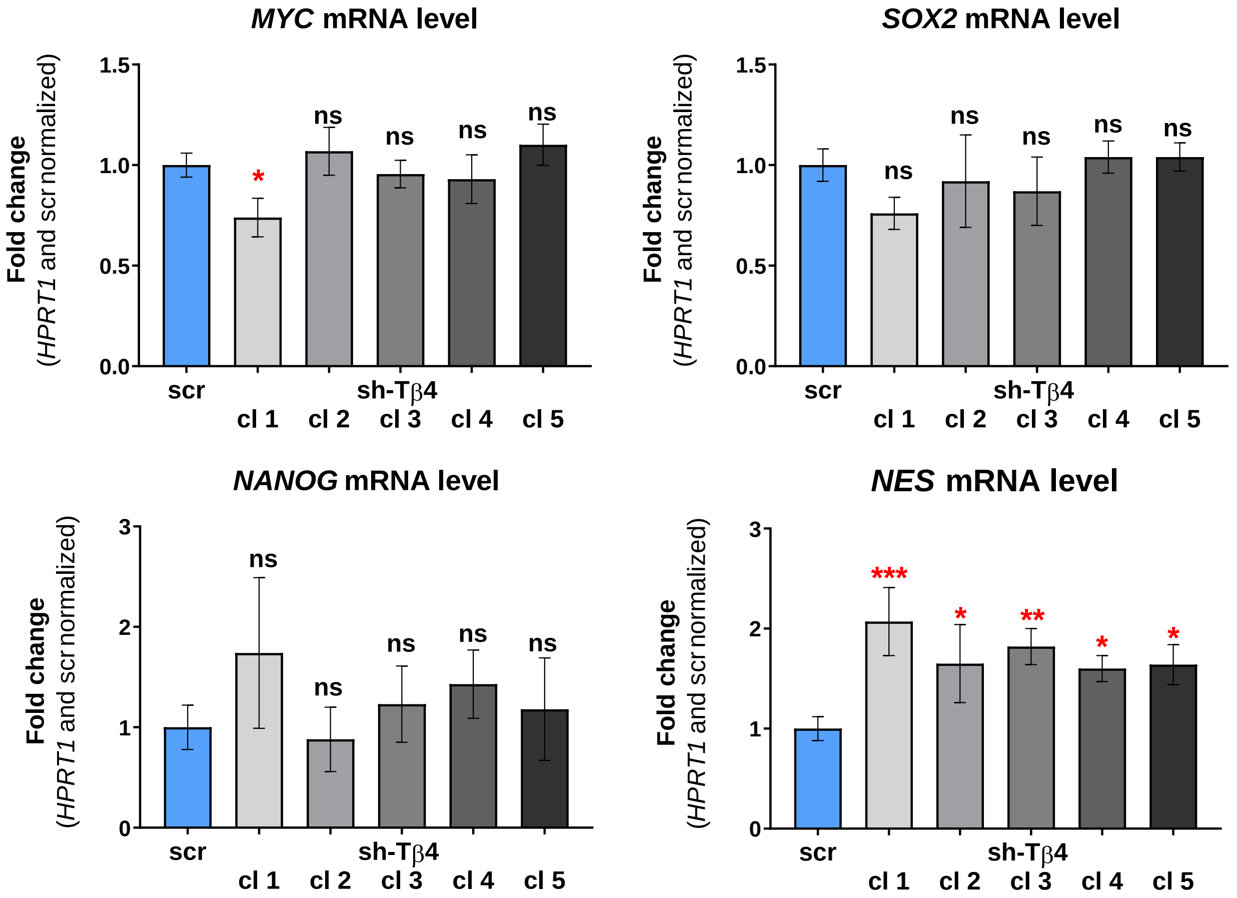

Figure S3. qPCR analysis of *MYC, SOX2, NANOG, NES* shown separately for each sh-Tβ4 clone. The results were normalized to the outcomes for *HPRT1* gene and scr (n =3). Graphs present the means±SD. The significance level was set at **P* < 0.05, ***P* < 0.01, ****P* < 0.001. This figure corresponds to the Figure 3C-F in the main text.

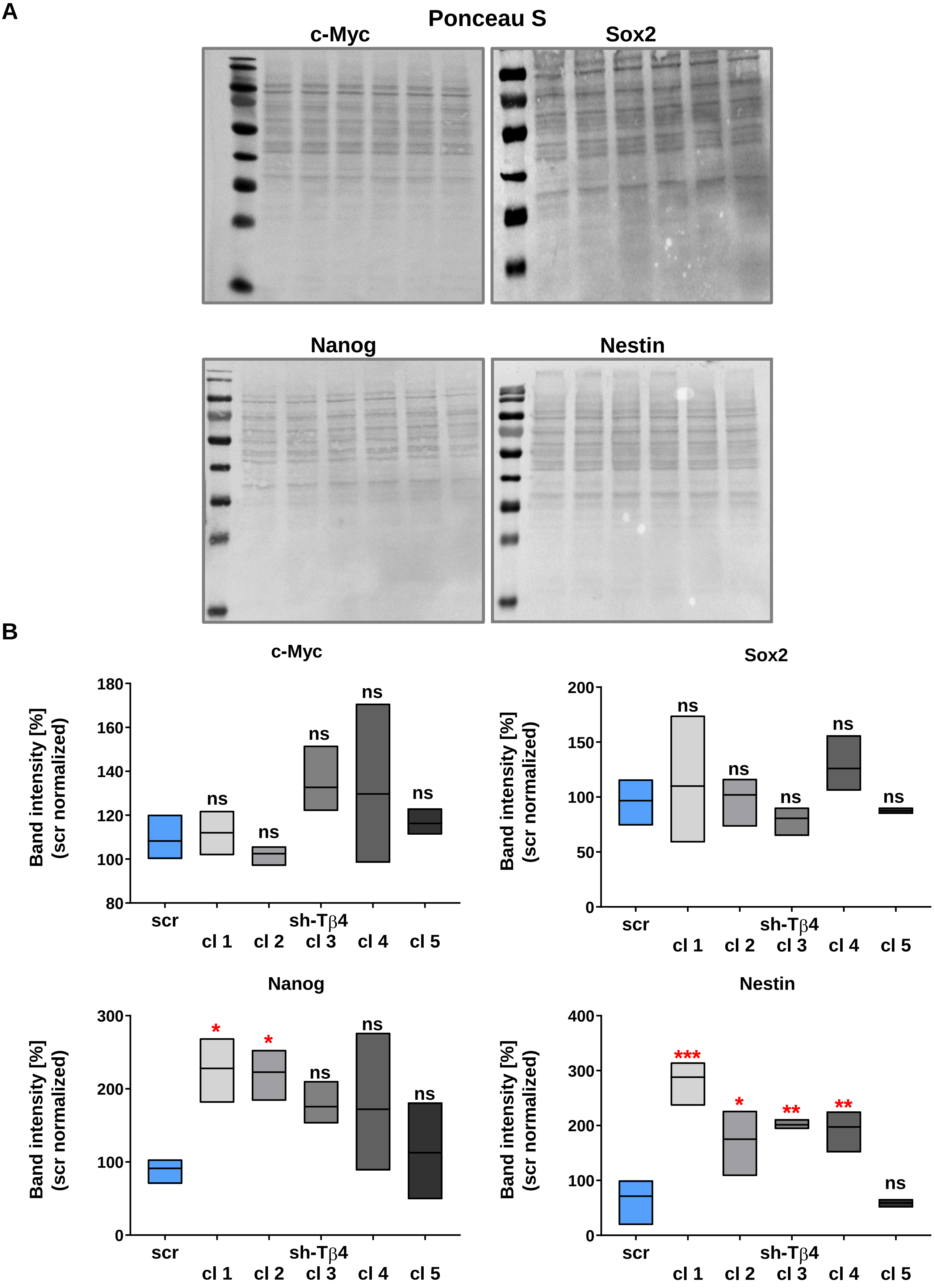

Figure S4. Evaluation of c-Myc, Sox2, Nanog and Nestin level shown separately for each sh-Tβ4 clone. (**A**) Ponceau S membrane stainings for total protein analysis (TPA) of immunoblots probed for: c-Myc, Sox2, Nanog, Nestin in scr and sh-Tβ4 clones shown in Figure 3G in the main text. (**B**) Densitometric analysis of c-Myc, Sox2, Nanog and Nestin Western blots NES shown separately for each sh-Tβ4 clone (n = 3). Graphs indicate average-max-min values. The significance level was set at *$P < 0.05$, **$P < 0.01$, ***$P < 0.001$. This subfigure corresponds to the Figure 3H in the main text.

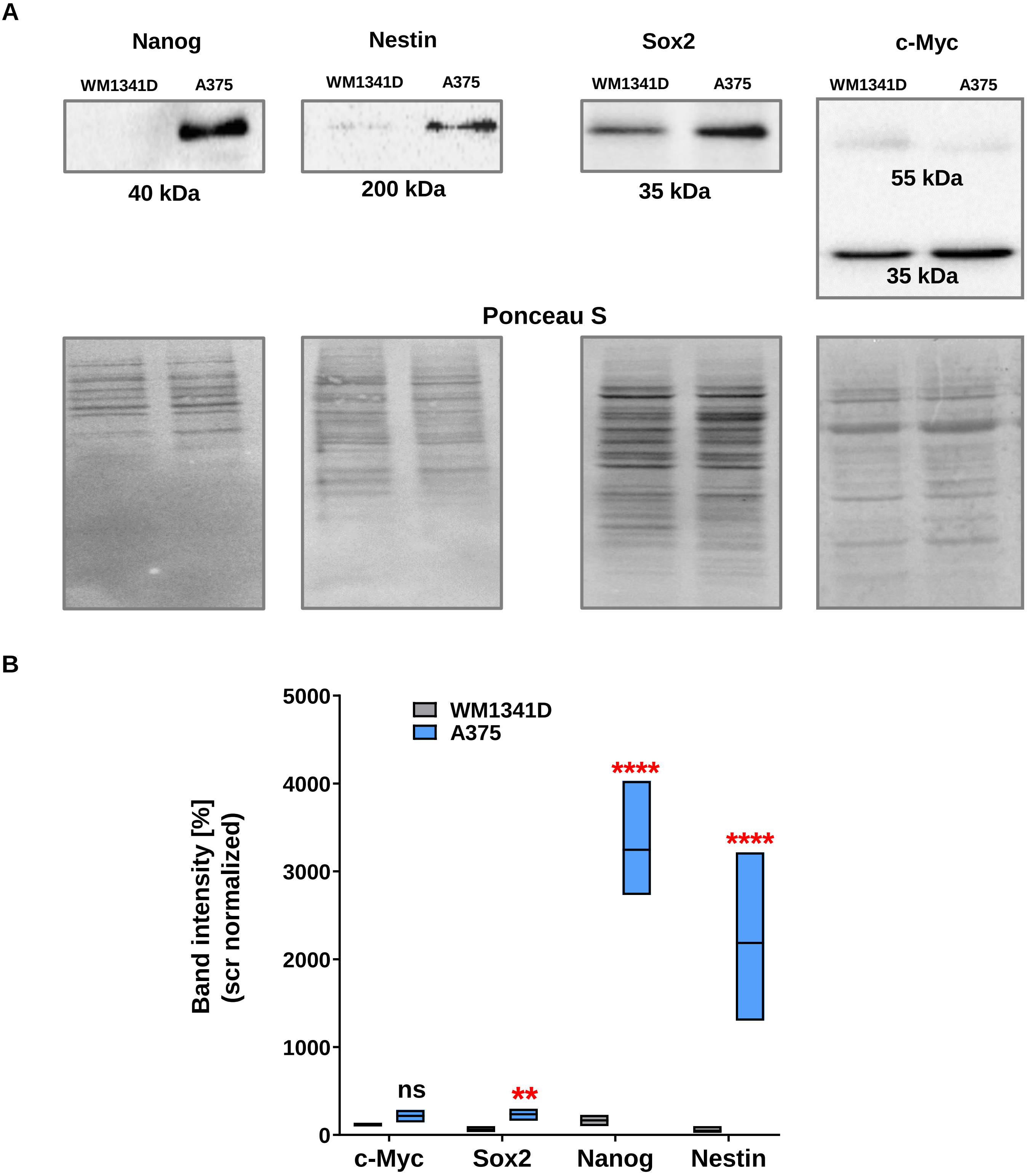

Figure S5. Evaluation of c-Myc, Sox2, Nanog and Nestin level in WM1341D and A375 cells. **(A)** Representative immunoblots of tested stemness-related markers and corresponding Ponceau S membrane stainings for WM1341D and A375 cells. 30 µg of protein was loaded on every lane. Membranes were probed for chosen stemness marker proteins: c-Myc, Sox2, Nanog and Nestin. **(B)** Densitometric analysis of c-Myc, Sox2, Nanog, and Nestin level in A375 and WM1341D cells (n = 3-4); The significance level was set at $^{**}P < 0.01$ or $^{****}P < 0.0001$. Graphs indicate average-max-min values.

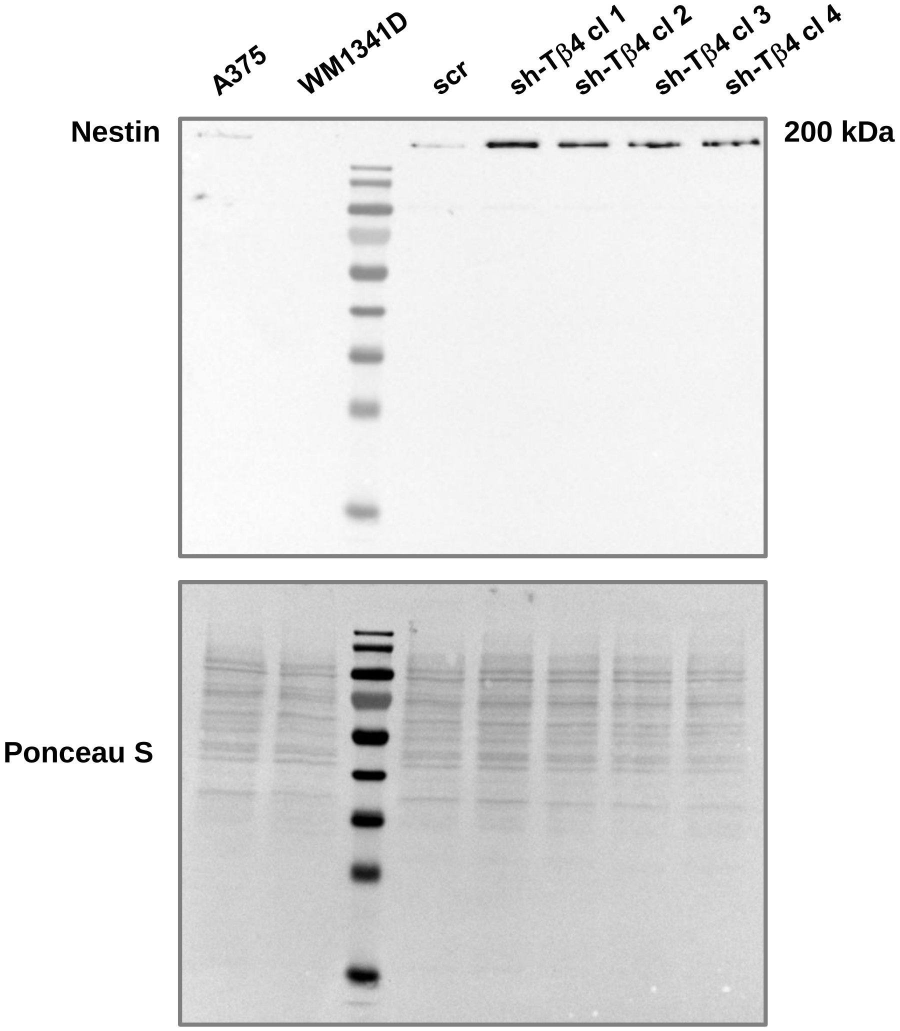

Figure S6. Evaluation of Nestin level in WM1341D and A375 cells, and scr and sh-Tβ4 clones. Lysates of listed cell types were analyzed on one nitrocelulose membrane. 30 μg of protein was loaded on every lane. Corresponding Ponceau S membrane stainings is also shown.

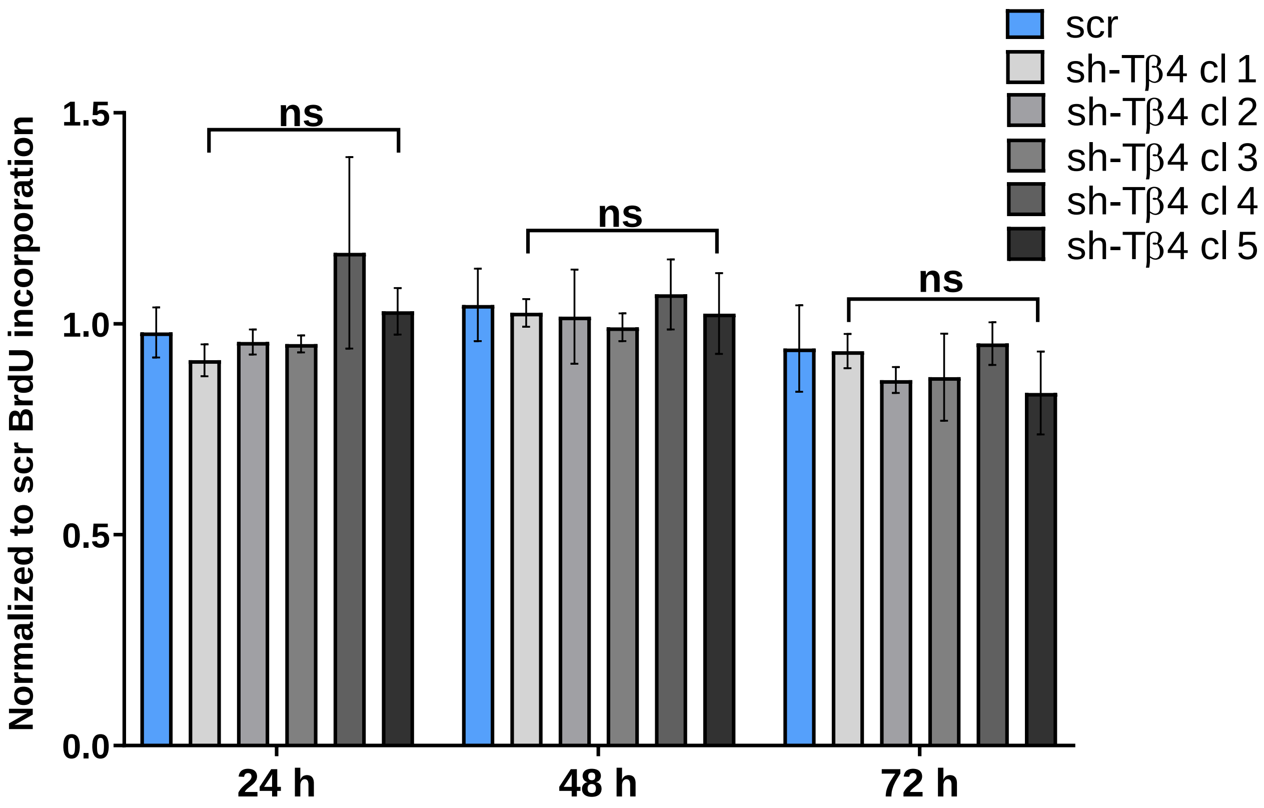

Figure S7. Analysis of cell proliferation rate shown separately for each sh-Tβ4 clone. Cell proliferation was measured using the BrdU assay 24, 48, and 72 h after seeding the cells (n = 3). The graph presents the mean±SD. The significance level was set at $^*P < 0.05$. This figure corresponds to the Figure 4B in the main text.

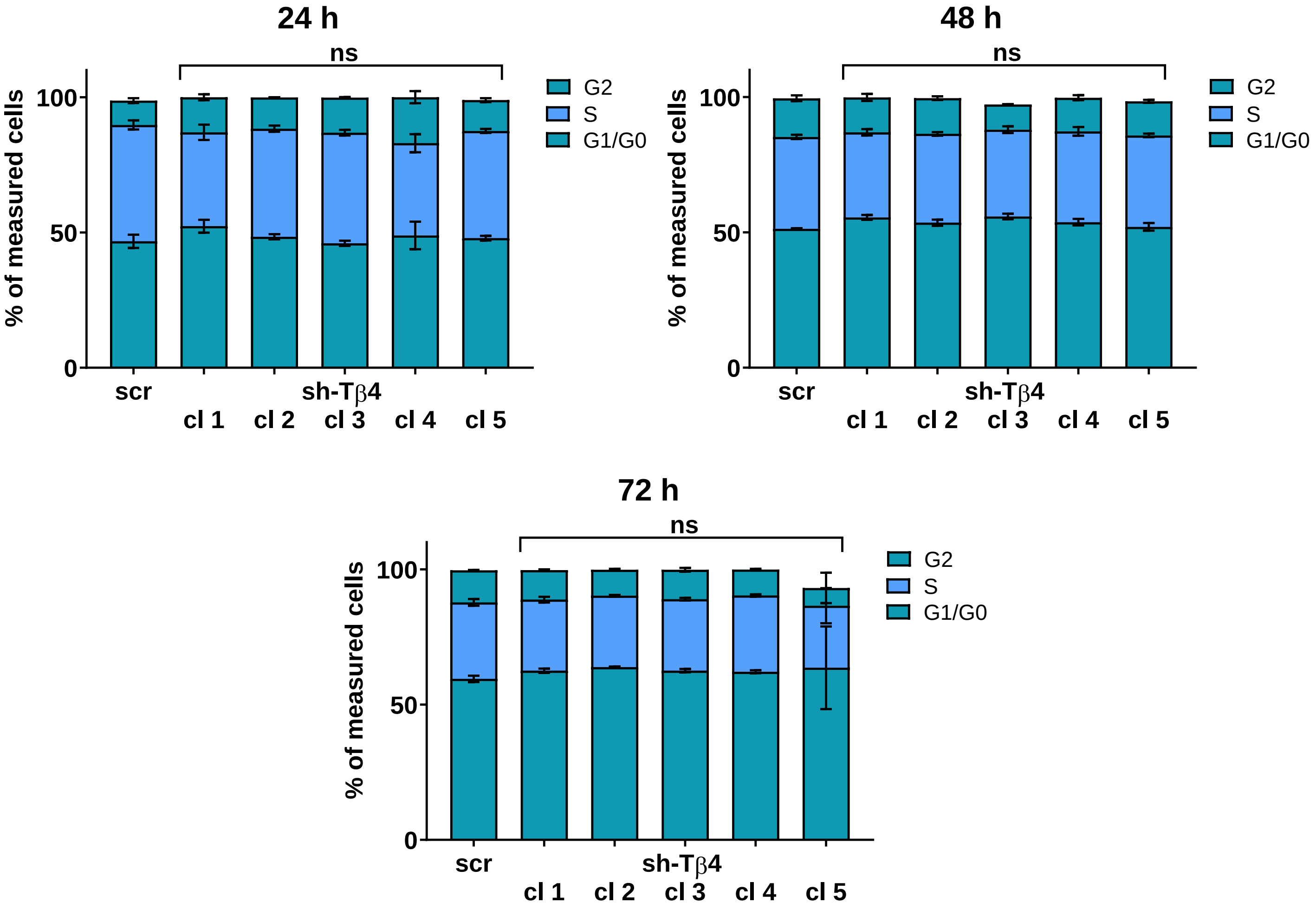

Figure S8. Analysis of cell cycle rate shown separately for each sh-Tβ4 clone. Analysis of the cell cycle was performed on scr and sh-Tβ4 cells collected respectively 24, 48, and 72 h after seeding (n = 3). The significance level was set at $^{*}P < 0.05$. This figure corresponds to the Figure 4F-H in the main text.

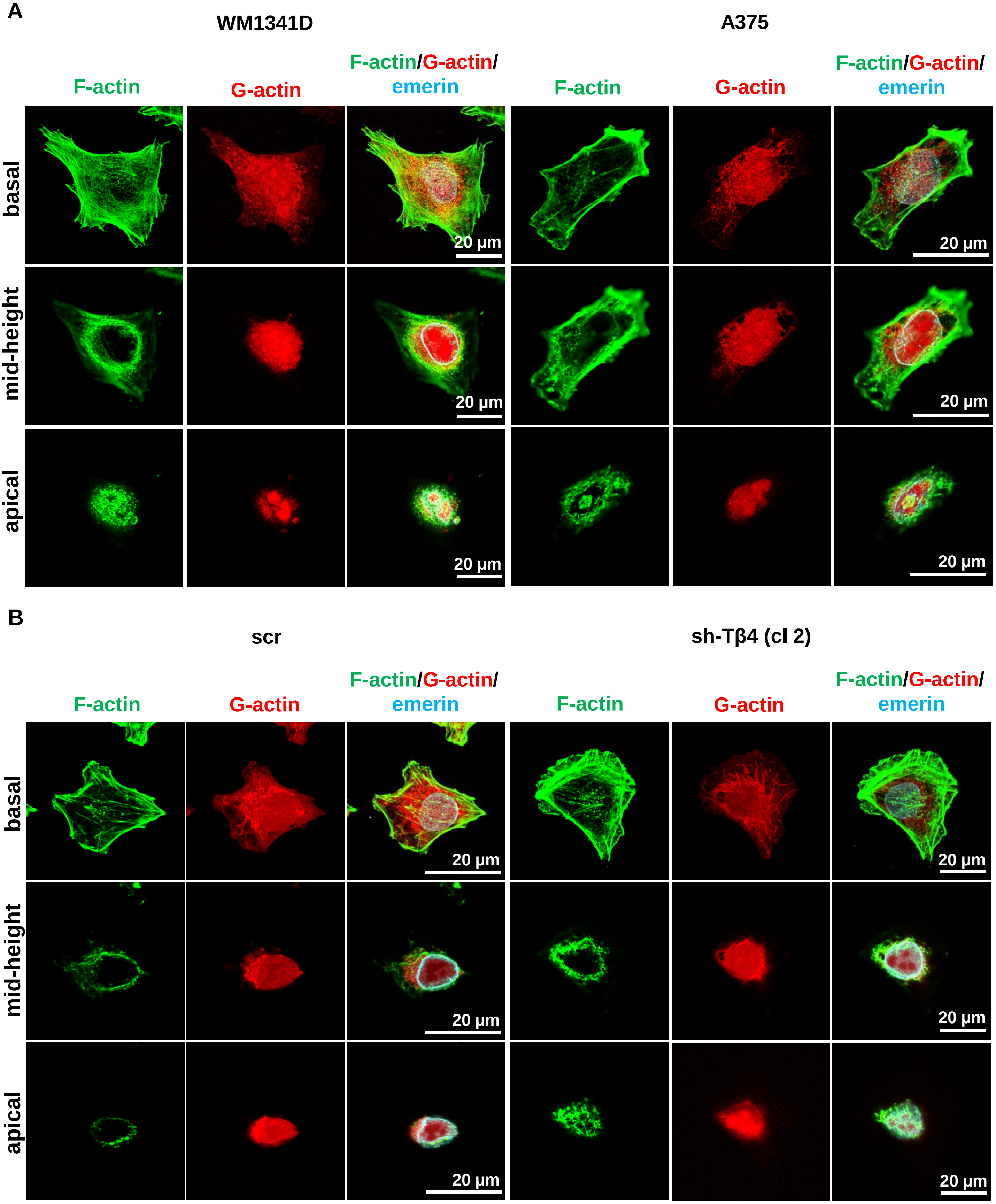
A
WM1341D
A375
F-actin
G-actin
F-actin/G-actin/emerin
basal
mid-height
apical
20 µm
B
scr
sh-Tβ4 (cl 2)
F-actin
G-actin
F-actin/G-actin/emerin
basal
mid-height
apical
20 µm

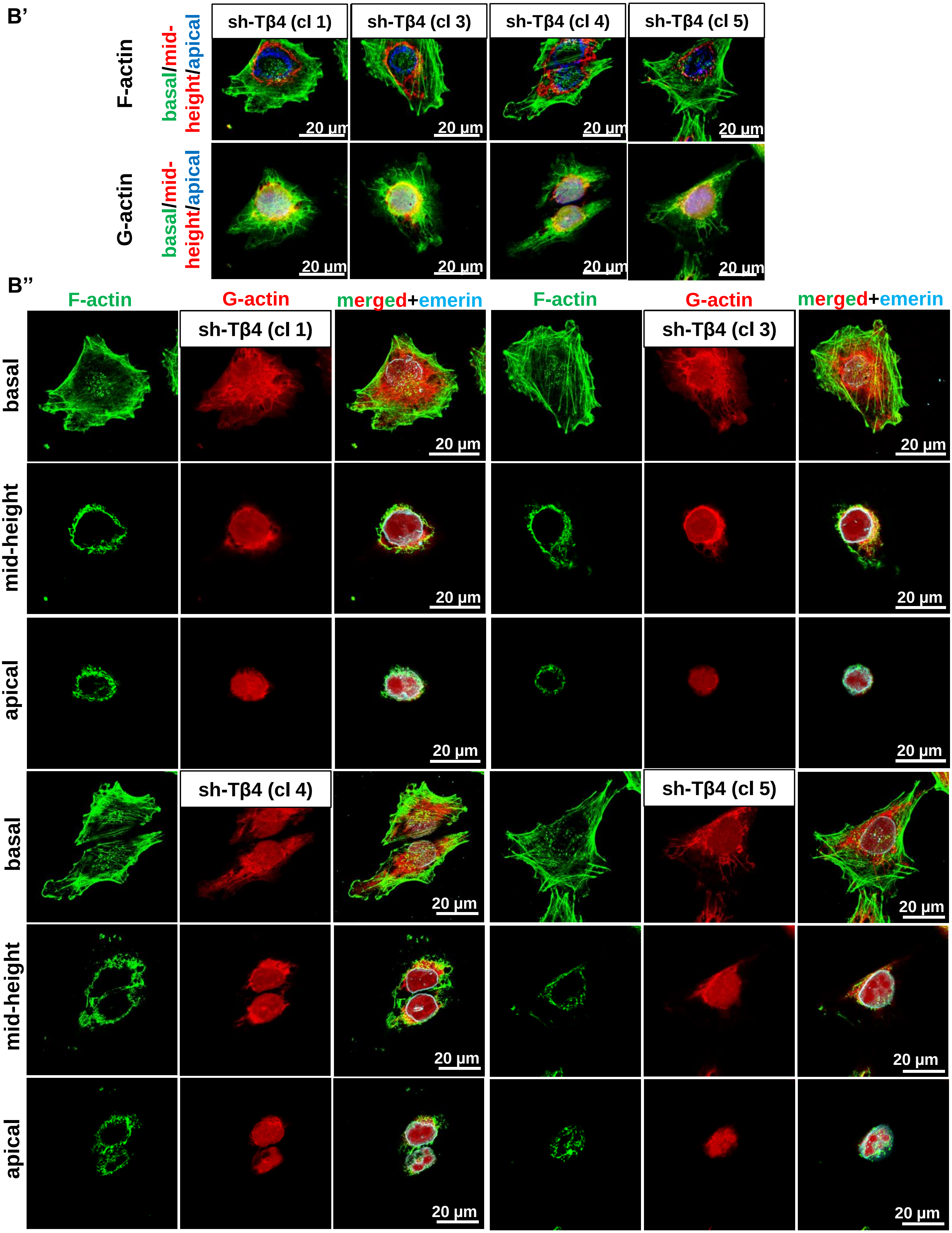

Figure S9. The role of Tβ4 in melanoma cells' actin cytoskeleton organization. Stainings to detect F-actin and G-actin in **(A)** WM1341D, A375 cells, and **(B-B'')** Scr and sh-Tβ4 clones. The confocal microscopic pictures for each type of cells were performed at three focal planes: cell contact area to the substratum, a cross-section at the cell nucleus, and nucleus apical outer surface. Additional staining was performed to detect nucleus envelope by using anti-emerin antibodies. This figure corresponds to the Figures 5 and 6 in the main text.

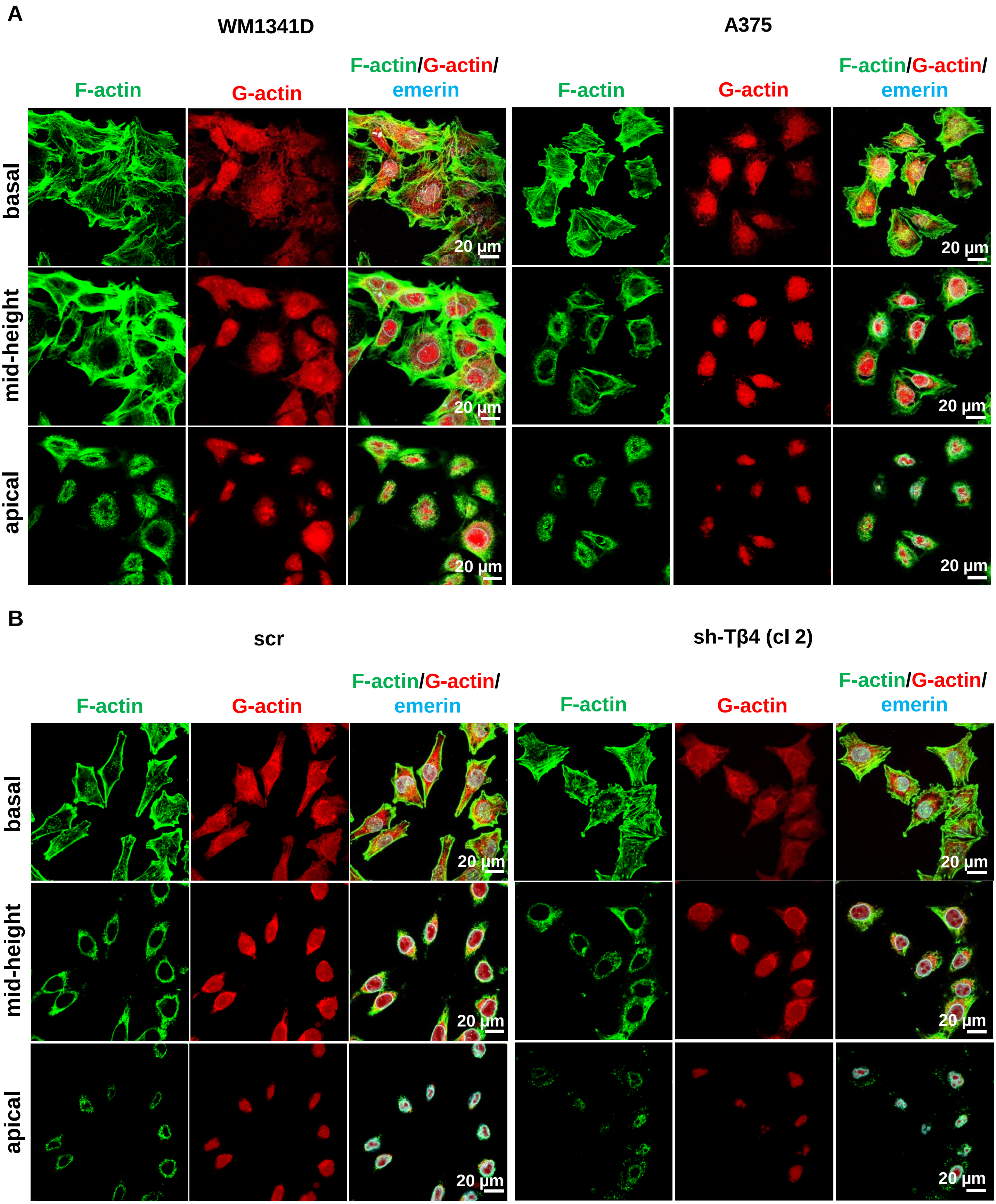
A
WM1341D
A375
F-actin
G-actin
F-actin/G-actin/
emerin
basal
mid-height
apical
20 µm
B
scr
sh-Tβ4 (cl 2)
F-actin
G-actin
F-actin/G-actin/
emerin
basal
mid-height
apical
20 µm

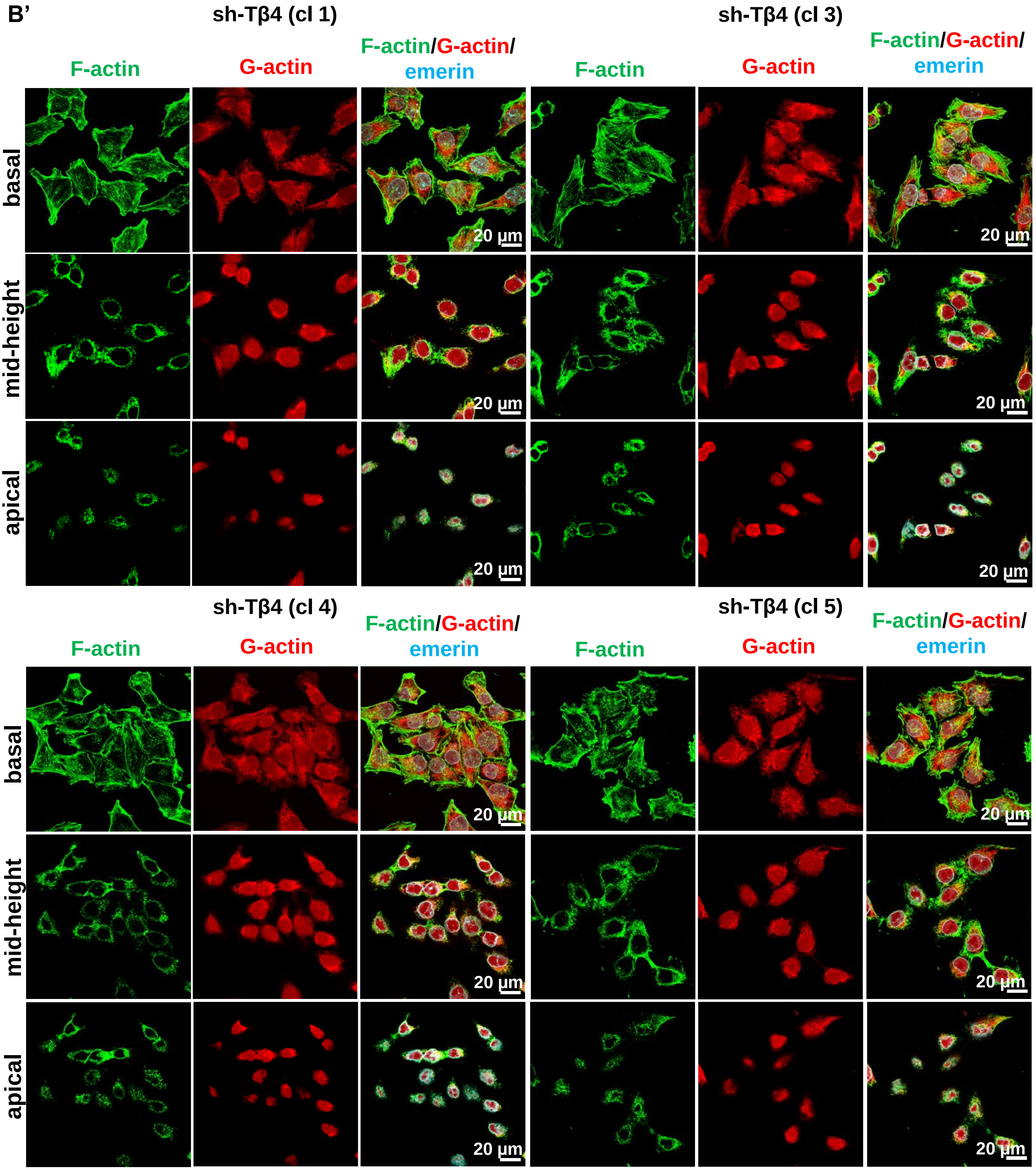

Figure S10. The role of Tβ4 in melanoma cells' actin cytoskeleton organization (cell population analysis). Stainings to detect F-actin and G-actin in **(A)** WM1341D, A375 cells, and **(B-B')** Scr and sh-Tβ4 clones. The confocal microscopic pictures for each type of cells were performed at three focal planes: cell contact area to the substratum, a cross-section at the cell nucleus, and nucleus apical outer surface. Additional staining was performed to detect nucleus envelope by using anti-emerin antibodies. This figure corresponds to the Figures 5 and 6 in the main text.

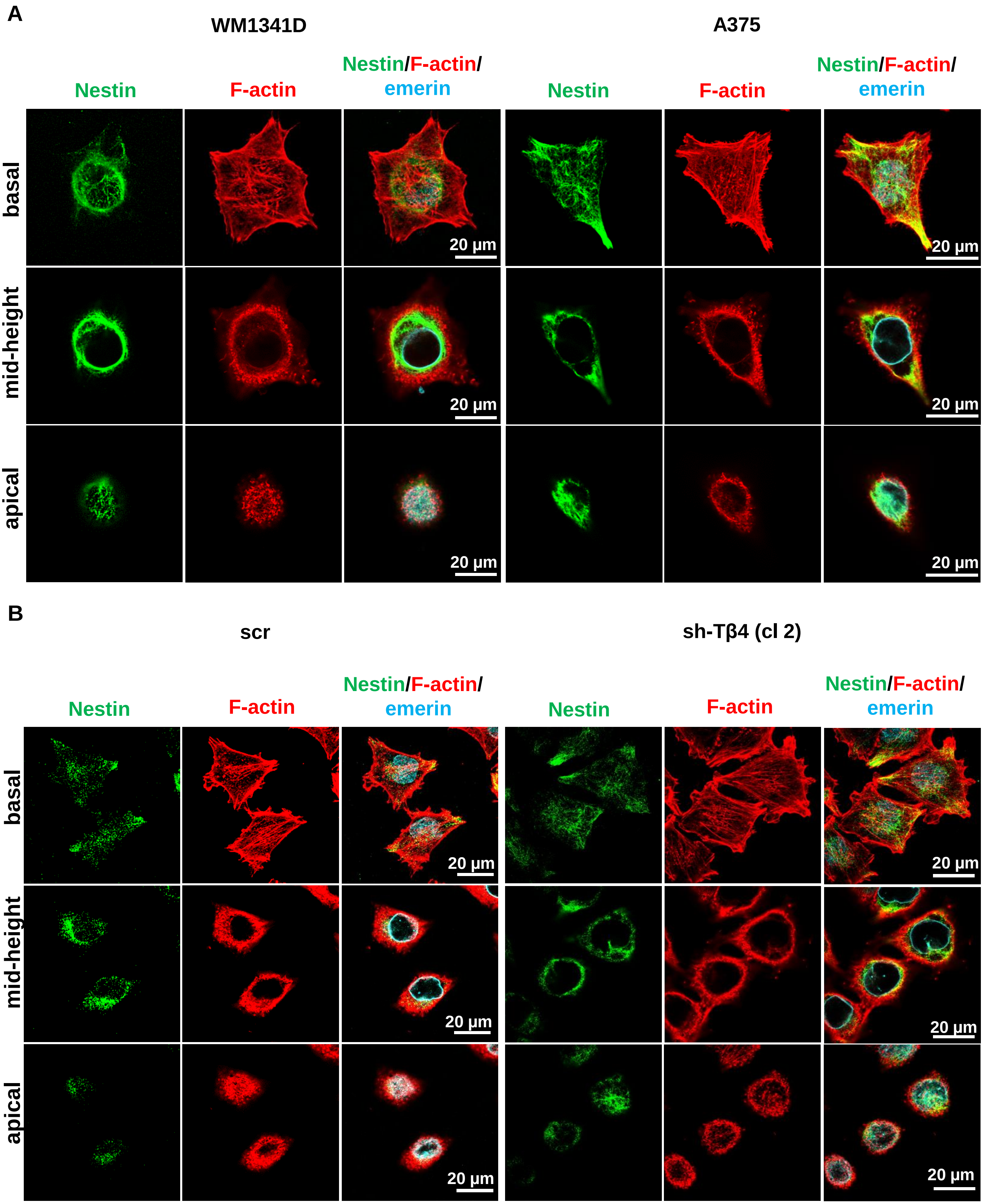
A
WM1341D
A375
Nestin
F-actin
Nestin/F-actin/emerin
basal
mid-height
apical
20 µm
B
scr
sh-Tβ4 (cl 2)
Nestin
F-actin
Nestin/F-actin/emerin
basal
mid-height
apical
20 µm

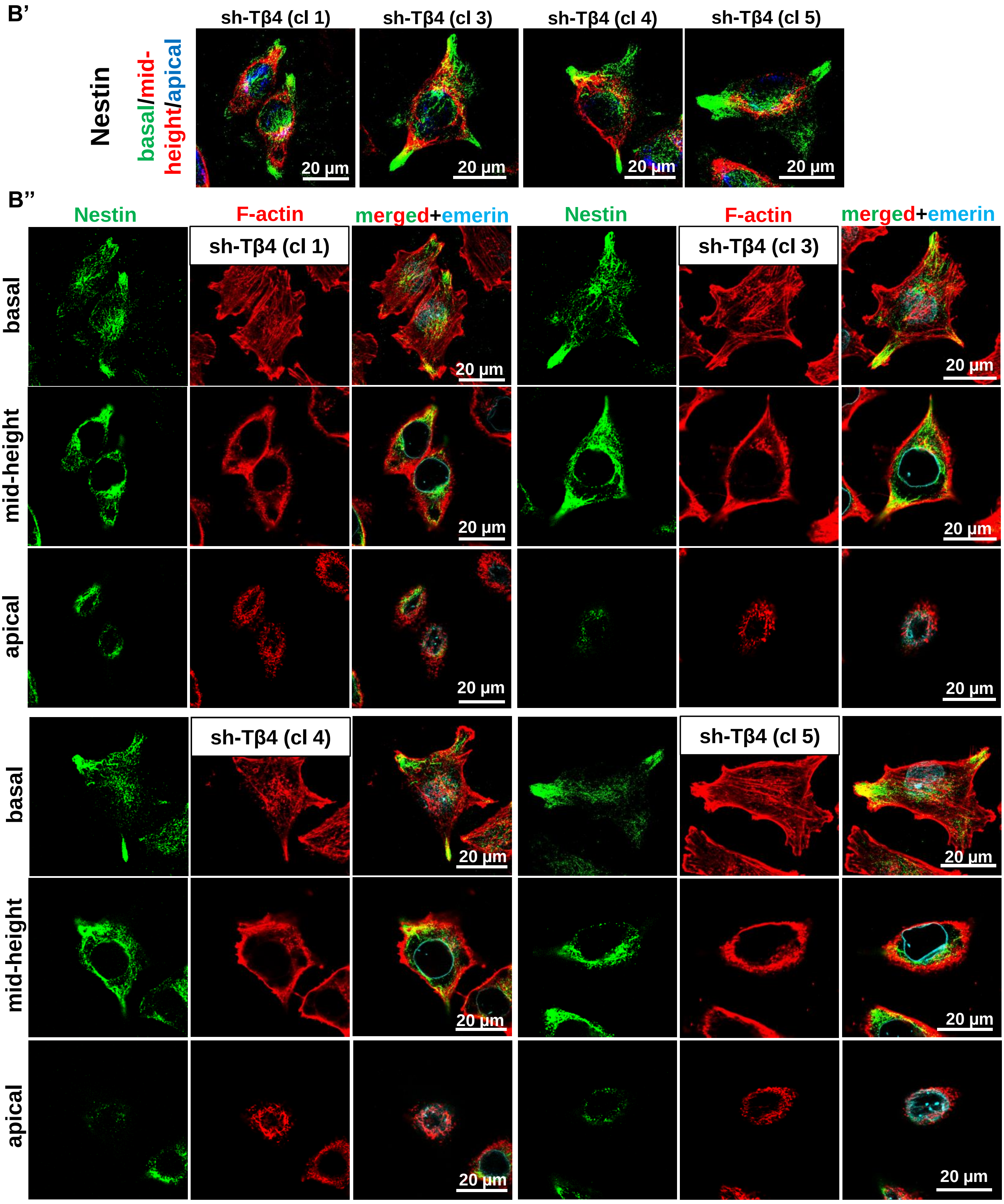

Figure S11. The role of Tβ4 in Nestin's organization in studied cells. Detection of nestin and F-actin in **(A)** WM1341D, A375 cells, and **(B-B'')** Scr and sh-Tβ4 clones was performed by application of appropriate antibodies and fluorescently labeled phalloidin, respectively. The confocal microscopic pictures for each type of cells were performed at three focal planes: cell contact area to the substratum, a cross-section at the cell nucleus, and nucleus apical outer surface. Additional staining was performed to detect nucleus envelope by using anti-emerin antibodies. This figure corresponds to the Figures 5 and 6 in the main text.

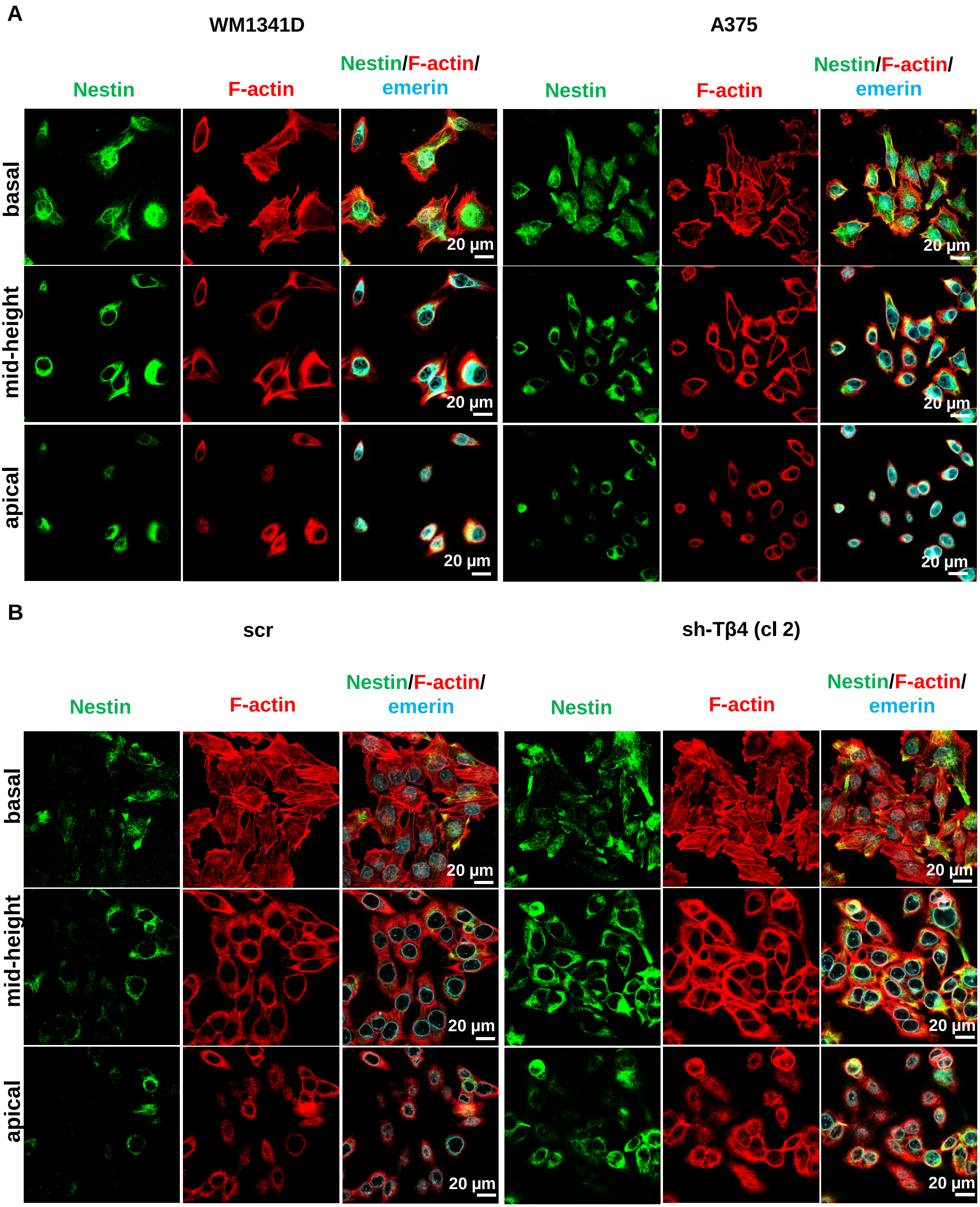
A
WM1341D
A375
Nestin
F-actin
Nestin/F-actin/emerin
basal
mid-height
apical
20 µm
B
scr
sh-Tβ4 (cl 2)

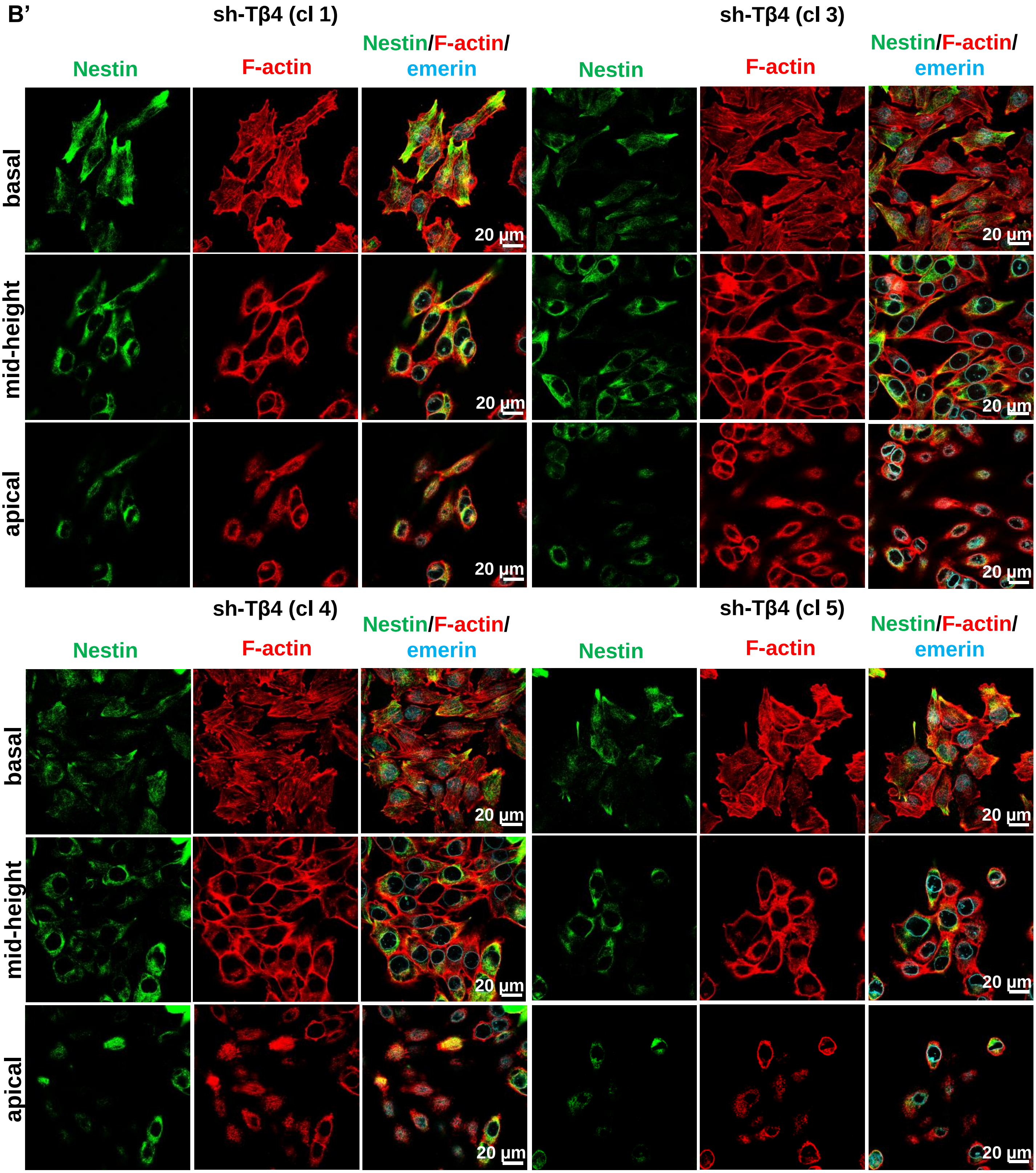

Figure S12. The role of Tβ4 in Nestin's organization in studied cells (cell population analysis). Detection of Nestin and F-actin in **(A)** WM1341D, A375 cells, and **(B-B')** Scr and sh-Tβ4 clones was performed by application of appropriate antibodies and fluorescently labeled phalloidin, respectively. The confocal microscopic pictures for each type of cells were performed at three focal planes: cell contact area to the substratum, a cross-section at the cell nucleus, and nucleus apical outer surface. Additional staining was performed to detect nucleus envelope by using anti-emerin antibodies. This figure corresponds to the Figures 5 and 6 in the main text.

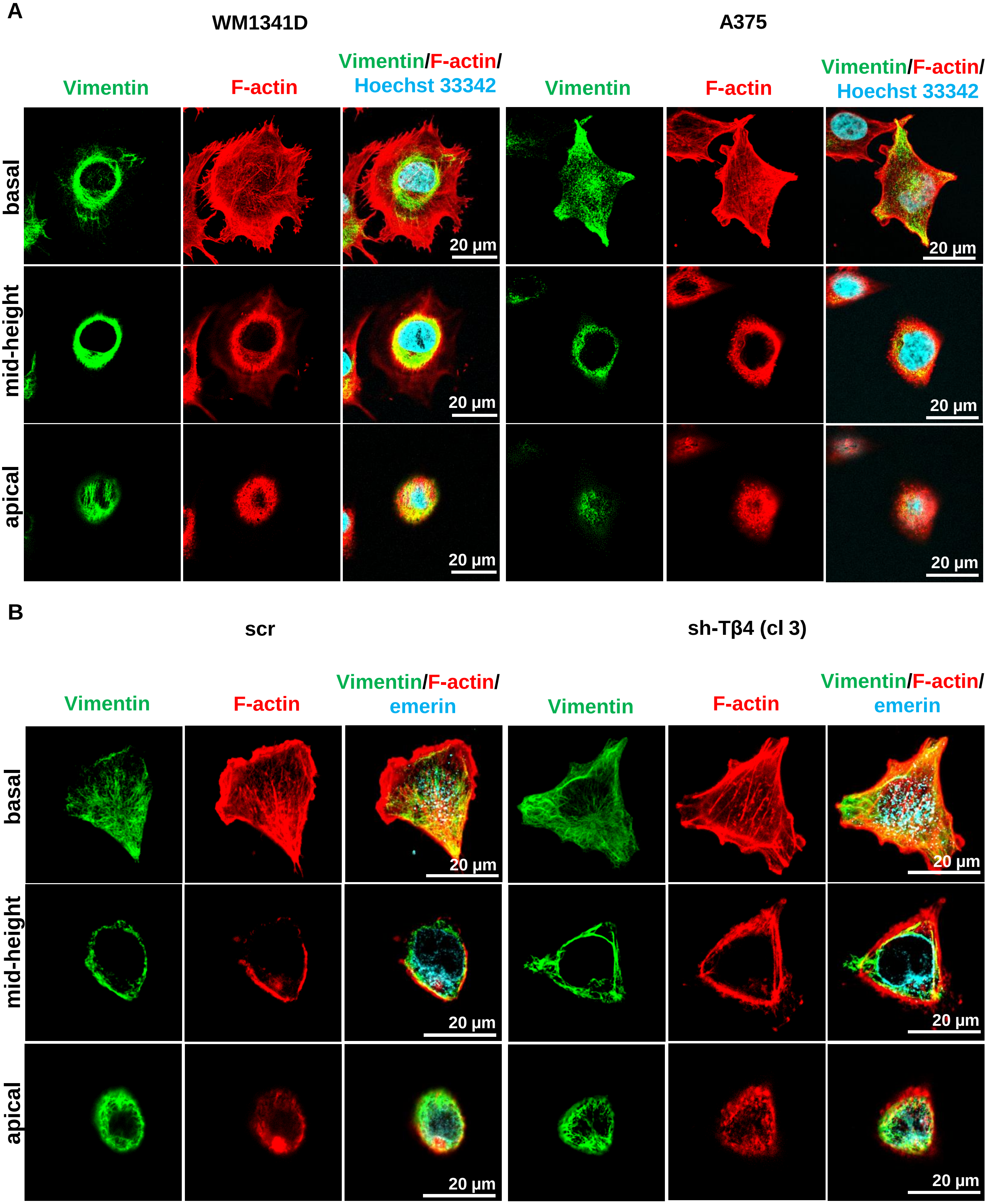
A
WM1341D
A375
Vimentin
F-actin
Vimentin/F-actin/
Hoechst 33342
Vimentin
F-actin
Vimentin/F-actin/
Hoechst 33342
basal
mid-height
apical
20 µm
B
scr
sh-Tβ4 (cl 3)
Vimentin
F-actin
Vimentin/F-actin/
emerin
Vimentin
F-actin
Vimentin/F-actin/
emerin
basal
mid-height
apical
20 µm

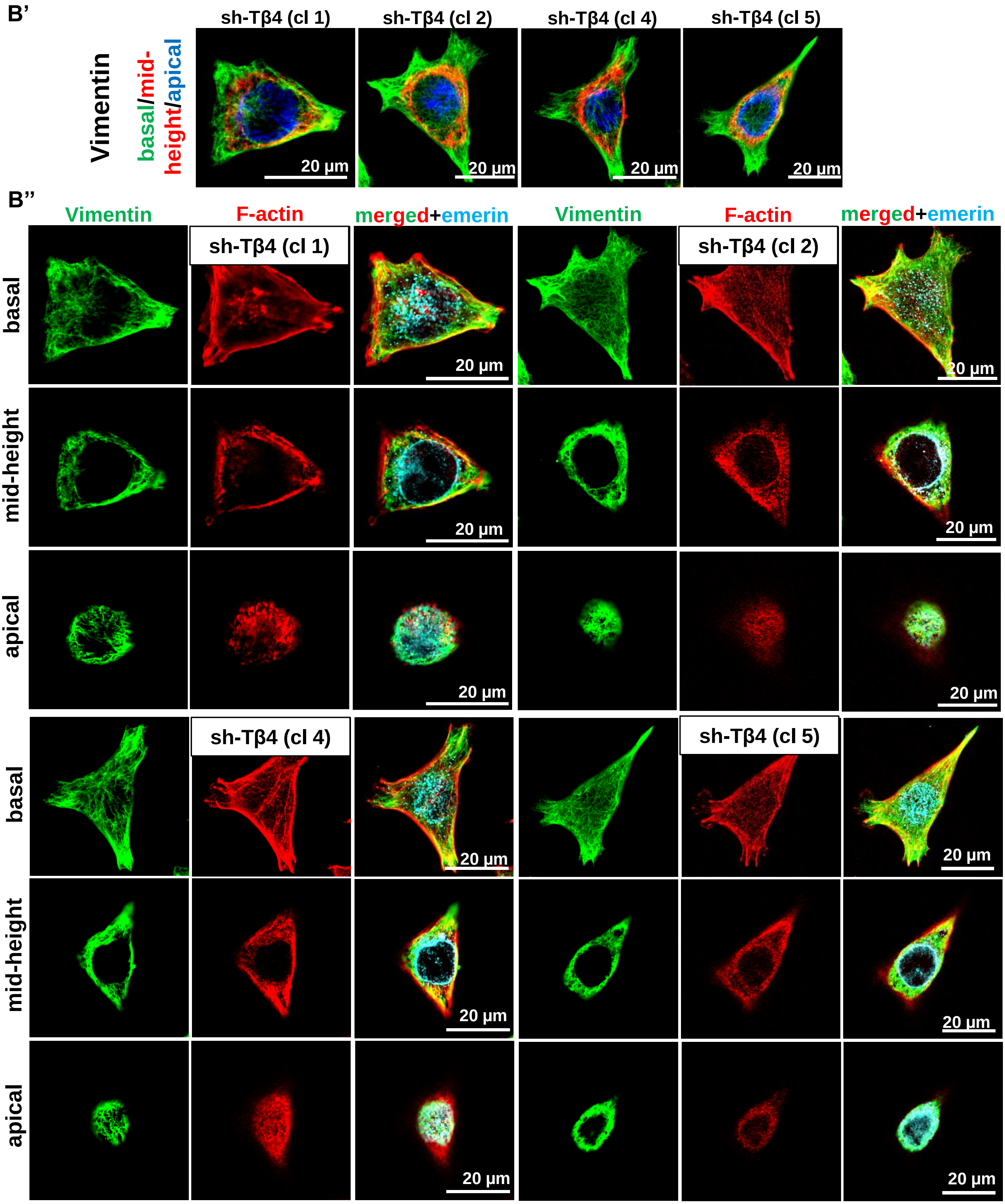

Figure S13. The role of Tβ4 in organization of Vimentin in melanoma cells. Immunocytochemical stainings to detect Vimentin in **(A)** WM1341D, A375 cells, and **(B-B'')** Scr and sh-Tβ4 clones. F-actin was detected by usage of fluorescently labeled phalloidin. The confocal microscopic pictures for each type of cells were performed at three focal planes: cell contact area to the substratum, a cross-section at the cell nucleus, and nucleus apical outer surface. Additional staining was performed to detect nucleus by using Hoechst 33342 dye or envelope by using anti-emerin antibodies. This figure corresponds to the Figures 5 and 6 in the main text.

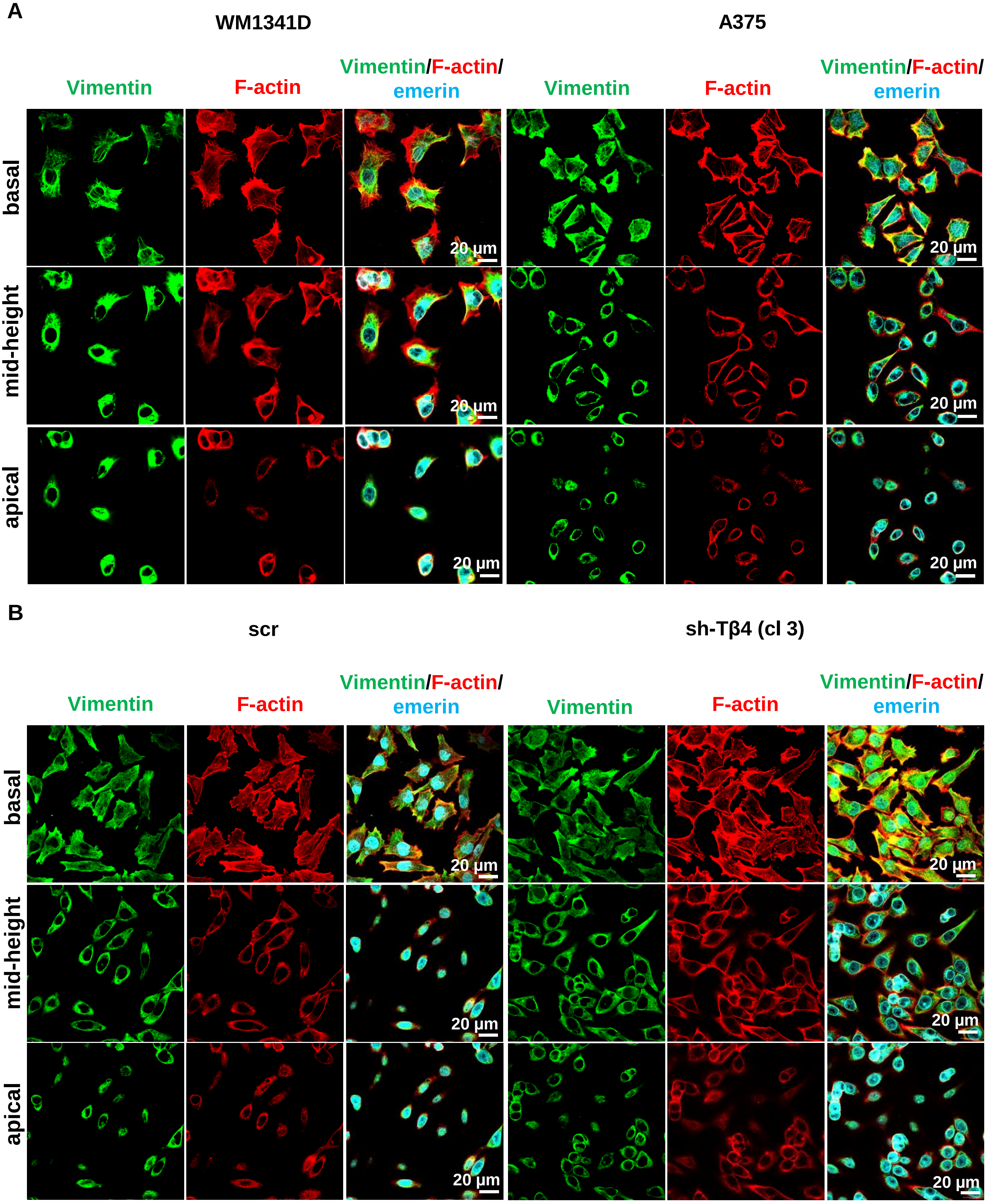

A
WM1341D
A375
Vimentin
F-actin
Vimentin/F-actin/emerin
basal
mid-height
apical
20 µm
B
scr
sh-Tβ4 (cl 3)
Vimentin
F-actin
Vimentin/F-actin/emerin
basal
mid-height
apical
20 µm

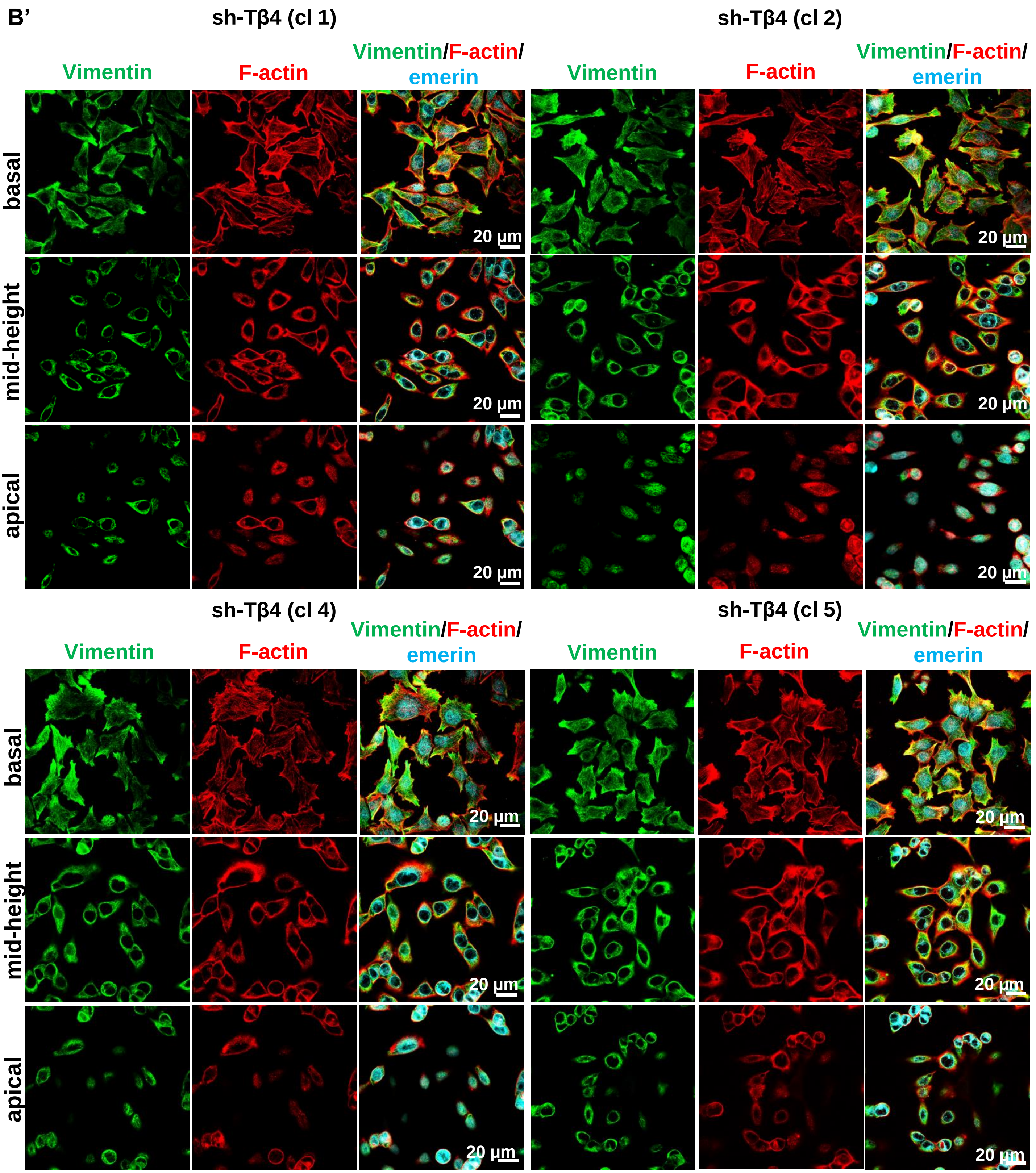

Figure S14. The role of Tβ4 in organization of Vimentin in melanoma cells (cell population analysis). Immunocytochemical stainings to detect Vimentin in **(A)** WM1341D, A375 cells, and **(B-B')** Scr and sh-Tβ4 clones. F-actin was detected by usage of fluorescently labeled phalloidin. The confocal microscopic pictures for each type of cells were performed at three focal planes: cell contact area to the substratum, a cross-section at the cell nucleus, and nucleus apical outer surface. Additional staining was performed to detect nucleus envelope by using anti-emerin antibodies. This figure corresponds to the Figures 5 and 6 in the main text.

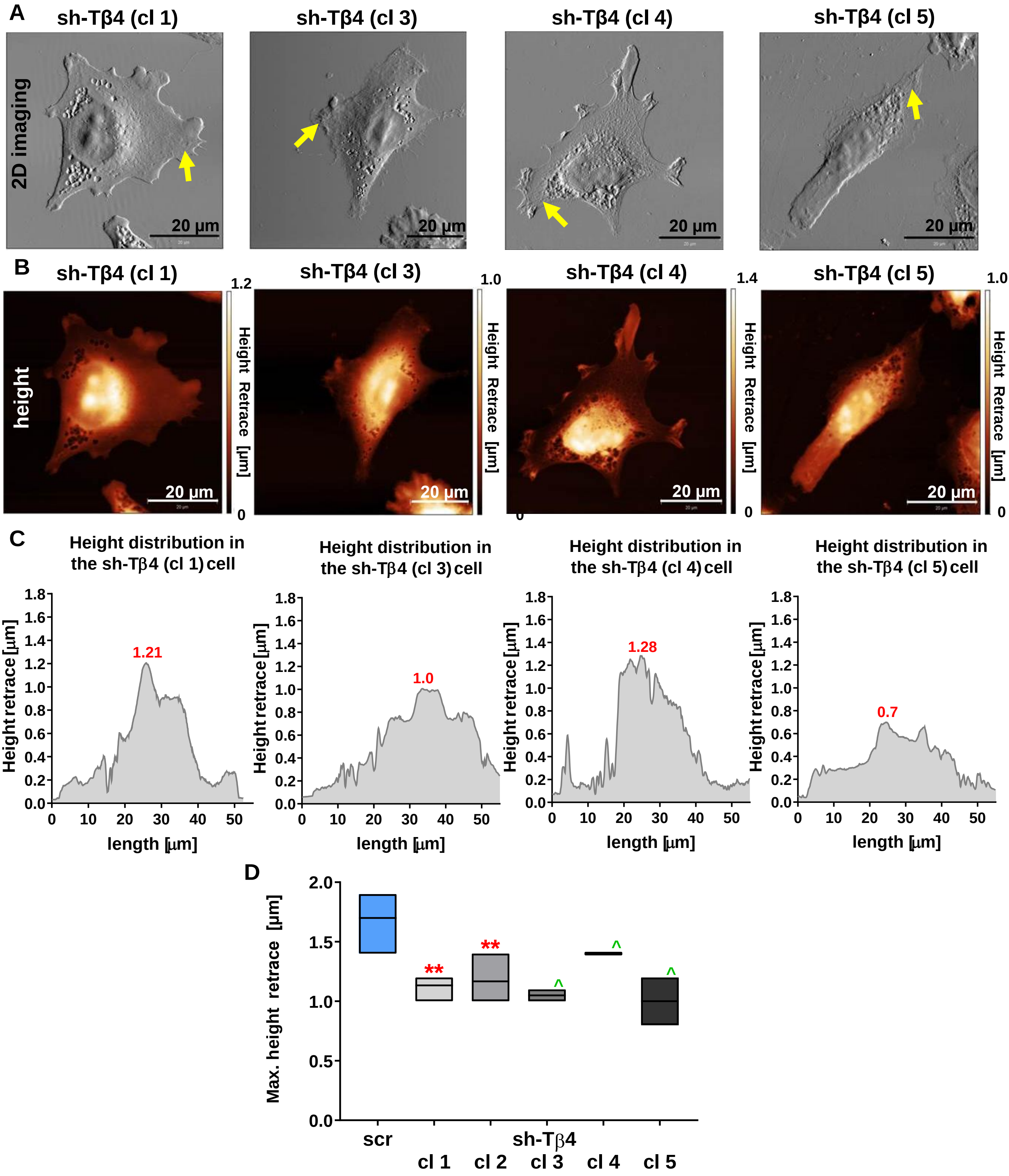

Figure S15. Analysis of the surface of sh-Tβ4 clones and their height. **(A)** Atomic force microscope (AFM) was used to analyze the surface of four A375 clones. Yellow arrows point at filamentous structures. **(B)** AFM analysis of body height presented in a form of a heat map (height retrace) of the cell body. **(C)** Histograms presenting the difference in the height of the cell in correlation to the position of a drawn line across the cell shown in A-B. **(D)** The bar chart presenting maximal height of the cells shown separately for each A375-sh*TMSB4X* clone. This figure corresponds to the Figure 7D in the main text. The graph indicates average-max-min values. The significance level was set at $^{**}P < 0.01$. ^ For these clones statistical analysis was not done as n=2.

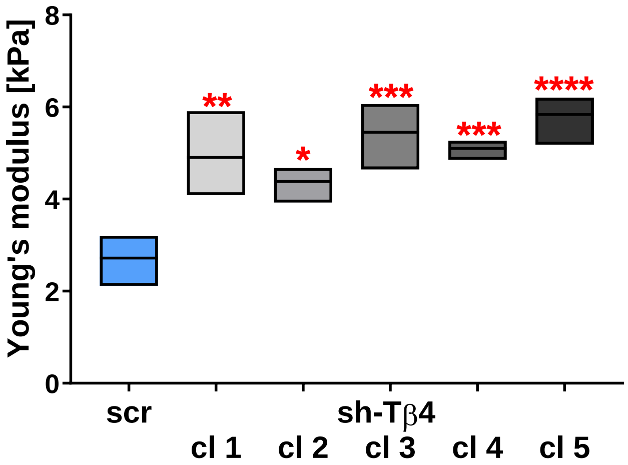

Figure S16. Single-cell force spectroscopy (SCFS) measurements shown separately for each sh-Tβ4 clone (n = 30). This figure corresponds to the Figure 8 in the main text. The graph indicates average-max-min values. The significance level was set at $*P < 0.05$, $**P < 0.01$, $***P < 0.001$, and $****P < 0.0001$.
